# Impact of heat shock transcription factor 1 on global gene expression profiles in cells which induce either cytoprotective or pro-apoptotic response following hyperthermia

**DOI:** 10.1186/1471-2164-14-456

**Published:** 2013-07-08

**Authors:** Małgorzata Kus-Liśkiewicz, Joanna Polańska, Joanna Korfanty, Magdalena Olbryt, Natalia Vydra, Agnieszka Toma, Wiesława Widłak

**Affiliations:** 1 Maria Skłodowska-Curie Memorial Cancer Center and Institute of Oncology, Gliwice Branch, Wybrzeże Armii Krajowej 15, Gliwice, Poland; 2 Biotechnology Centre for Applied and Fundamental Sciences, Department of Biotechnology, University of Rzeszow, Sokołowska St. 26, 36-100, Kolbuszowa, Poland; 3 Institute of Automatic Control, The Silesian University of Technology, Gliwice, Poland

**Keywords:** Spermatogenesis, Heat shock response, Gene expression profiles, Transcription factor binding, Apoptosis

## Abstract

**Background:**

Elevated temperatures induce activation of the heat shock transcription factor 1 (HSF1) which in somatic cells leads to heat shock proteins synthesis and cytoprotection. However, in the male germ cells (spermatocytes) caspase-3 dependent apoptosis is induced upon HSF1 activation and spermatogenic cells are actively eliminated.

**Results:**

To elucidate a mechanism of such diverse HSF1 activity we carried out genome-wide transcriptional analysis in control and heat-shocked cells, either spermatocytes or hepatocytes. Additionally, to identify direct molecular targets of active HSF1 we used chromatin immunoprecipitation assay (ChIP) combined with promoter microarrays (ChIP on chip). Genes that are differently regulated after HSF1 binding during hyperthermia in both types of cells have been identified. Despite HSF1 binding to promoter sequences in both types of cells, strong up-regulation of *Hsps* and other genes typically activated by the heat shock was observed only in hepatocytes. In spermatocytes HSF1 binding correlates with transcriptional repression on a large scale. HSF1-bound and negatively regulated genes encode mainly for proteins required for cell division, involved in RNA processing and piRNA biogenesis.

**Conclusions:**

Observed suppression of the transcription could lead to genomic instability caused by meiotic recombination disturbances, which in turn might induce apoptosis of spermatogenic cells. We propose that HSF1-dependent induction of cell death is caused by the simultaneous repression of many genes required for spermatogenesis, which guarantees the elimination of cells damaged during heat shock. Such activity of HSF1 prevents transmission of damaged genetic material to the next generation.

## Background

Somatic cells protect themselves from damage during cellular stress inducing so called heat shock response, which results in heat shock proteins (HSPs) synthesis. There are many different HSPs classified to families based on molecular weight and sequence homology. In mammalian cells there are five major classes of HSP families, namely the HSPH (HSP110), HSPC (HSP90), HSPA (HSP70), DNAJ (HSP40) and HSPB (small HSP exemplified by HSP27), and two chaperonin families: HSPD/E (HSP60/HSP10) and CCT (TRiC) [1]. Generally, HSPs prevent inappropriate protein aggregation and mediate transport of immature proteins to the target organelles for final packaging, degradation or repair. Although some members of HSPs are expressed constitutively in the absence of stress or during gametogenesis and embryogenesis [2,3], many of them accumulate to a high level in cells subjected to different types of stress stimuli and remain elevated for a prolonged period [4]. Heat shock factor 1 (HSF1) is the primary transcription factor responsible for the activation of *Hsp* genes following stress. In physiological conditions HSF1 exists as an inactive monomer. Activation of HSF1 in response to cellular stress is connected with its trimerization, phosphorylation and binding to DNA in the promoter regions containing the heat shock elements (HSEs), which are present mainly in heat shock genes [5]. In addition to the regulation of *Hsp* genes, HSF1 is involved in the transcription of numerous other genes, both in the absence or presence of heat shock. In *Saccharomyces* or *Drosophila* the direct transcriptional targets of HSF represent nearly 3% of genes [6,7]. These genes encode for proteins involved in diverse cellular processes such as RNA splicing, apoptosis, ubiquitinylation and protein degradation, detoxification, energy generation, carbohydrate metabolism, small molecule transport, cell signaling and maintenance of cell integrity [6-8].

Despite the high degree of conservation of the heat shock response, different cells vary in their ability to induce HSPs synthesis and consequently in sensitivity to damaging agents. HSPs overexpression in various human cancers diminishes the success of anti-cancer treatment by increasing the resistance of cancer cells to therapy [9]. On the other hand, some neurons, pre-ovulatory oocytes, spermatocytes and some stages of embryonic development [10-13], as well as certain tumor cell lines (especially of myeloid origin, e.g. lymphomas; [14]), are hypersensitive to elevated temperatures. It has been shown, at least for spermatocytes, that inducible HSP70 expression is blocked in such cells [15,16]. Opposite to most somatic cells, in which HSF1 is a part of the cytoprotective system, in spermatocytes it acts as a pro-apoptotic factor [17,18]. Moreover, the testis-specific variant of HSP70 is depleted in cells undergoing HSF1-induced apoptosis [19]. Activation of HSF1 in male germ cells induces massive degeneration of a seminiferous epithelium, which leads to male infertility [16-18]. In fact, primary spermatocytes are germ cells the most sensitive to heat stress [13]. Spermatocytes are very unique cells. They originate from spermatogonia and divide by meiosis giving haploid spermatids (that finally differentiate to spermatozoa) [20]. During the whole process of spermatogenesis dramatic changes in patterns of gene expression and chromatin structure are observed. In particular, the first meiotic division (occurring in primary spermatocytes) involves many cell-specific gene products. They are needed for correct processing of chromosome condensation, pairing of homologous chromosomes, formation of the synaptonemal complexes, and genetic recombination. These unique processes should be highly orchestrated and any disturbances at that stage of spermatogenesis could lead to fertility problems [21].

In the majority of mammals, the male gonads are located outside the main body cavity to provide the lower testicular temperature required for correct spermatogenesis and fertility. Increasing of the testis temperature up to the body temperature (or above it) leads to the activation of HSF1 [22]. Active HSF1 acts as a cell-survival factor only in pre-meiotic germ cells, but not in meiotic and post-meiotic ones [15]. Both mitochondria-dependent and death receptor-dependent pathways appear to be involved in the HSF1-induced apoptosis of spermatogenic cells: the levels of BCL-2 family proteins is increased, p53 protein accumulates and expression levels of caspase-8 and death-receptor-interacting proteins (including FADD and TRADD) are elevated [16]. The apoptosis of spermatocytes induced by HSF1 can be an important mechanism involved in the removal of aberrant germ cells. This can prevent maturation of damaged male germ cells and transmission of incorrect genetic information to the next generation.

Transcriptional activity of HSF1 is apparently indispensable in its pro-apoptotic functions within spermatocytes. However, details of molecular mechanisms involved in activation of HSF1-triggered apoptosis of male germ cells are not known at present. We performed studies that aimed to disclose the initiating events leading to apoptosis of male germ cells after temperature elevation. For this purpose, the genome-wide transcriptional analysis was performed both in control and heat-shocked cells, either isolated mouse spermatocytes or hepatocytes representing somatic cells, using the Affymetrix GeneChip system. Genes that are differently expressed after hyperthermia in both types of cells have been identified. To find out genes directly regulated by HSF1, the chromatin immunoprecipitation assay combined with DNA microarray (ChIP on chip) was performed. This approach enabled the identification of genes targeted by this transcription factor, in either somatic or male germ cells.

## Results

### Differences in transcriptional response to hyperthermia between somatic and spermatogenic cells

**Figure 1:**
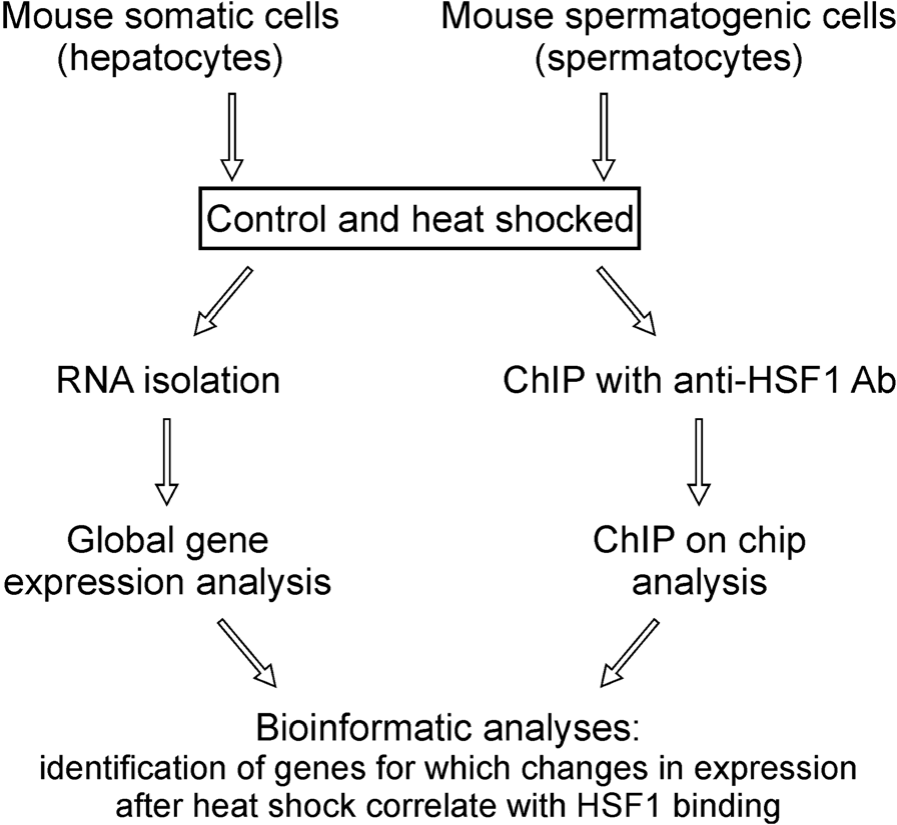
General scheme of the study.

**Figure 2:**
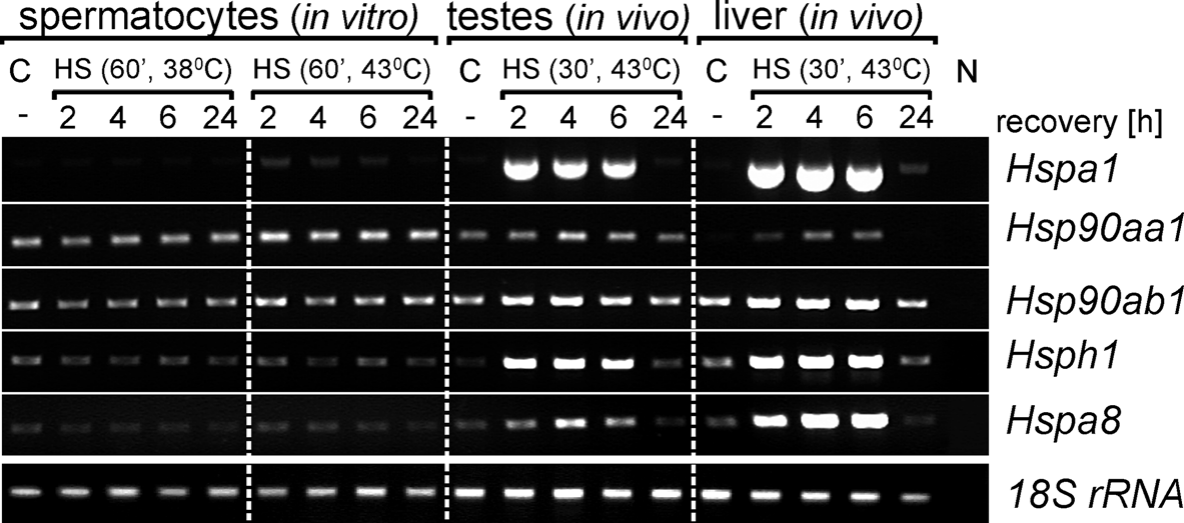
**Monitoring of the heat shock response in isolated spermatocytes, testes and liver.** Transcripts of selected *Hsp* genes up to 24 h after hyperthermia were detected by RT-PCR. Loading control reactions were performed with primers specific to *18S rRNA* transcript; N, PCR negative control without template.

The physiological temperature for mouse testes (normally located outside the body cavity) is approximately 32-33°C. Increasing the testicular temperature up to 37°C and above does not induce cytoprotective mechanisms, as it is observed in somatic cells during hyperthermia, but leads to the degeneration of seminiferous epithelium [13]. Aiming to decipher the differences in molecular mechanisms induced by heat shock in mouse somatic and spermatogenic cells we applied the strategy which is schematically illustrated in Figure 1. In the first step, we performed the global gene expression profiling using Affymetrix microarrays in control and heat shocked cells. As model somatic cells we used hepatocytes that respond to hyperthermia in a classical way by induction of heat shock genes transcription (Figure 2). As spermatogenic cells we used a fraction of cells enriched with spermatocytes (isolated by unit gravity sedimentation in bovine serum albumin, BSA, gradient), which are the most sensitive to damage at elevated temperatures [13]. Using isolated spermatocytes we avoided the influence of the somatic testicular component in the final results. We took advantage of *Hsp* genes as reference genes to monitor response to heat shock, and observed strong induction of *Hspa1*, *Hspa8* and *Hsph1* genes transcription two to six hours after hyperthermia in the liver and in whole testes, but not in the fraction of isolated spermatocytes (Figure 2). This result is consistent with previous findings that mRNAs of major heat shock genes are hardly induced *in vivo* in response to heat shock in male germ cells [15,23]. On Affymetrix gene chip arrays we analyzed RNA isolated from untreated (control) hepatocytes and after 2 h of recovery at the physiological temperature from heat shock performed at 43°C. In the case of spermatocytes, RNA was isolated from untreated cells and after 2 h of recovery at the physiological temperature from heat shock performed at 38°C or 43°C.

To exclude from analyses genes with very low levels of expression thresholds of signals (in arbitrary units in log2) were calculated (Additional file 1: Table S1 [24]). Expression levels of 15 364 genes with signals above the noise threshold level registered in at least one experimental point are presented in Additional file 2[24]. Changes in the level of genes expression in heat-shocked (HS) samples versus control, untreated (C), are presented as a signal log ratio (SLR; the average value of HS/average value of C). For each experimental group significant SLR was calculated, and genes affected by hyperthermia in spermatocytes and hepatocytes were found (Table 1). The analysis revealed that global changes in the levels of expression in spermatocytes were smaller (not exceeding 8-fold changes up or down) than in hepatocytes (exceeding 64-fold up). However, in spermatocytes subjected to hyperthermia at 38°C many more genes were affected than in hepatocytes (Table 1). Importantly, while in hepatocytes the number of up- and down-regulated genes was comparable, in spermatocytes significantly more genes were inhibited than up-regulated.

**Table 1:** Representation of genes changing their expression following heat shock

	**Induction**		**Repression**	
	**Range of SLR (significant to max.)**	**Number of genes [% of total]**	**Range of SLR (min. to significant)**	**Number of genes [% of total]**
Spermatocytes HS_38°C versus control	0.82 to 2.21	517 [3.53]	−2.84 to −0.82	909 [6.20]
Spermatocytes HS_43°C versus control	0.49 to 2.43	18 [0.12]	−1.35 to −0.36	362 [2.47]
Hepatocytes HS_43°C versus control	0.75 to 6.17	155 [1.07]	−3.62 to −0.75	133 [0.92]

Changes of expression are shown as SLRs. “Total” includes all genes with signal above noise at least in one experimental point (14 653 genes in spermatocytes and 14 420 in hepatocytes).

The ten top genes with the highest induction of the transcription after hyperthermia are shown in Additional file 3: Table S2 [24]. Approximately 30% of genes activated in spermatocytes subjected to heat shock at 38°C (SLR ≥ 0.82) were expressed in hepatocytes at a very low level (below the noise threshold). In turn, almost 40% encode for proteins with an unknown function. Cluster analysis and the *Gene to GO BP* (Gene Ontology Biological Process) test showed that, among activated genes, those involved in negative regulation of different biological processes are over-represented (Additional file 4; clusters: 87, 79 [24]). Importantly, activation of gene expression was rather marginal in spermatocytes subjected to heat shock at 43°C (Table 1 and Additional file 5[24]). Genes that were induced in hepatocytes following hyperthermia (SLR ≥ 0.75) were usually not up-regulated in spermatocytes, and for 20% of them the level of expression in spermatocytes was below the noise threshold. The noted exception was activation of *Hspa1* gene in both hepatocytes and spermatocytes (after heat shock at 43°C), which suggests that the isolated fraction of spermatogenic cells might be slightly contaminated by somatic cells. As expected, the *Gene to GO BP* test for over-representation revealed that genes grouped in clusters with the highest degree of induction in hepatocytes are involved mainly in processes corresponding to the stress response and protein folding. Strongly activated genes are also associated with inflammatory response, cytokine production and T cell activation (Additional file 6; clusters: 1, 6, 21, 65, 16, 25, 47 [24]).

The ten top genes with the highest inhibition of the transcription following hyperthermia are shown in Additional file 7: Table S3 [24]. Among genes down-regulated in spermatocytes following heat shock at 38°C (SLR ≤ −0.82) a large portion (6.4%) was not expressed in hepatocytes, and uncharacterized genes represented about 10%. Cluster analysis and the *Gene to GO BP* test showed that among genes down-regulated in spermatocytes at 38°C strongly over-represented are those involved in RNA metabolism, cellular process involved in reproduction, regulation of gene expression and signal transduction (Additional file 4; clusters: 22, 55, 11, 82, 16, 83, 7, 15 [24]). In spermatocytes subjected to hyperthermia at 43°C inhibition of the transcription was more prominent than activation, although it was weaker than in cells treated at 38°C. Down regulation of genes involved in response to heat and response to oxidative stress was the most characteristic (Additional file 5; clusters: 34, 50 [24]). Genes down-regulated in heat shocked hepatocytes (SLR ≤ −0.75) are involved in lipid, steroid and isoprenoid metabolism, catabolic processes and oxidation-reduction processes (Additional file 6; clusters: 48, 30, 97, 6 [24]); 30% of such genes showed very low level of expression in spermatocytes (below the noise threshold). It should be also noted that for genes with the highest repression following the hyperthermia of hepatocytes the big differences in the level of expression between samples were observed (Additional file 7: Table S3, part C [24]), which makes this group less conclusive.

Our expression data indicated that responses of spermatocytes to hyperthermia at 38°C and at 43°C were quantitatively and qualitatively different. It was previously suggested that heat shock at 43°C resulted rather in rapid changes of the protein localization than their levels (e.g. BAX and cytochrome c redistribution was observed in spermatocytes shortly after treatment; [25]). Hence, comparison of changes induced in hepatocytes treated at 43°C with changes induced in spermatocytes treated at 38°C appeared more relevant (in both cases the temperature of the hyperthermia was 5-6°C above physiological).

Both hepatocytes and spermatocytes showed significant differences in the transcriptional response to stress, and only a few genes responded similarly (Additional file 8: Table S4 [24]). The majority of genes affected by hyperthermia were differentially regulated in hepatocytes and spermatocytes. As expected, *Hsp* genes and some other genes coding for proteins that enable recovery from stress were strongly induced in hepatocytes, but not in spermatocytes. Furthermore, some *Hsp* genes were strongly down-regulated in spermatocytes, although there were also examples of chaperones’ expression activation (Table 2).

**Table 2:** The most significant changes in expression of genes from the HSPs family (and some other chaperones)

**Entrez gene ID**	**Mean SC_C**	**Mean SC_38**	**SLR SC_38 vs C**	**Mean HEP_C**	**Mean HEP_43**	**SLR HEP_43 vs C**	**Gene symbol (full name)**
1100037258	7.01	5.48	**−1.53**	9.34	9.13	−0.22	*Dnajc3* (DnaJ (Hsp40) homolog, subfamily C, member 3)
12330	8.69	7.54	**−1.15**	10.29	10.07	−0.22	*Canx* (calnexin)
15502	9.50	8.41	**−1.09**	11.65	12.72	**1.07**	*Dnaja1* (DnaJ (Hsp40) homolog, subfamily B, member 1)
22027	10.93	9.90	**−1.03**	11.78	11.63	−0.15	*Hsp90b1* (heat shock protein 90, beta (Grp94), member 1)
224273	9.90	8.87	**−1.02**	5.95	5.92	−0.03	*Crybg3* (beta-gamma crystallin domain containing 3)
14231	7.16	8.75	**1.59**	5.51	5.61	0.10	*Fkbp7* (FK506 binding protein 7)
53380	6.92	8.25	**1.33**	6.83	6.77	−0.06	*Psmd10* (proteasome (prosome, ma-cropain) 26S subunit, non-ATPase, 10)
70430	6.94	8.07	**1.12**	6.87	6.96	0.09	*Tbce* (tubulin-specific chaperone E)
193740	5.86	6.46	0.59	6.93	13.10	**6.17**	*Hspa1a* (heat shock protein 1A)
81489	11.40	10.78	−0.62	8.48	12.88	**4.40**	*Dnajb1* (DnaJ (Hsp40) homolog, subfamily B, member 1)
58233	8.23	8.79	0.56	6.18	9.98	**3.80**	*Dnaja4* (DnaJ (Hsp40) homolog, subfamily A, member 4)
15511	4.96	5.03	0.07	9.55	13.22	**3.67**	*Hspa1b* (heat shock protein 1B)
15505	7.77	7.64	−0.13	9.18	11.65	**2.47**	*Hsph1* (heat shock 105kDa/110kDa protein 1)
80888	5.09	5.32	0.23	10.16	11.73	**1.58**	*Hspb8* (heat shock protein 8)
67035	10.05	10.21	0.16	8.83	10.19	**1.36**	*Dnajb4* (DnaJ (Hsp40) homolog, subfamily B, member 4)
12406	5.78	6.21	0.43	6.36	7.72	**1.36**	*Serpinh1* (serine (or cysteine) peptidase inhibitor, clade H, member 1)
15516	9.94	9.35	−0.59	11.29	12.31	**1.02**	*Hsp90ab1* (heat shock protein 90 alpha (cytosolic), class B member 1)
15528	7.03	7.09	0.06	8.63	9.64	**1.01**	*Hspe1* (heat shock protein 1 (chaperonin 10))

The level of expression in spermatocytes (SC) and in hepatocytes (HEP) is given in arbitrary units in logarithmic scale (log2). Changes in gene expression following heat shock (at 38°C or 43°C) are shown as SLRs.

Expression of several genes encoding proteins involved (or potentially involved) in cell death was differentially regulated in both types of cells (Table 3). Genes coding for proteins with proved anti-apoptotic properties (e.g. *Bag3*, *Phlda1*) were strongly activated only in hepatocytes. In spermatocytes some genes involved in regulation of apoptosis were repressed (e.g. inhibitors of apoptosis: *Api5* and *Syvn1*). It is noteworthy that expression of *Pyhin1,* which possibly promote ubiquitination and subsequent degradation of MDM2 leading to TP53 stabilization [26], was observed only in spermatocytes. This is consistent with the observation that p53 was accumulated in the testes of transgenic mice expressing active HSF1 [16].

**Table 3:** The most significant changes in expression of genes involved in cell death regulation

**Entrez gene ID**	**Mean SC_C**	**Mean SC_38**	**SLR SC_38 vs C**	**Mean HEP_C**	**Mean HEP_43**	**SLR HEP_43 vs C**	**Gene symbol (full name)**	**Function**
76747	6.80	8.31	**1.51**	4.82	4.48	−0.33	*Dapl1* (death associated protein-like 1)	Role in the early stages of epithelial differentiation or in apoptosis
236312	6.99	8.16	**1.17**	5.30	5.33	0.03	*Pyhin1* (pyrin and HIN domain family, member 1)	Promotes ubiquitination and subsequent degradation of MDM2
234463	8.55	9.68	**1.13**	7.70	7.88	0.18	*Tmem184c* (transmembrane protein 184C)	Inhibitor of cell growth
50996	7.67	6.00	**−1.68**	5.56	5.95	0.39	*Pdcd7* (programmed cell death 7)	May be selectively involved in specific apoptotic processes in mouse T-cells
75736	8.89	7.25	**−1.64**	6.35	6.17	−0.18	*Bcl2l12* (BCL2-like 12 (proline rich)	Potent apoptotic inhibitor
11800	7.85	6.32	**−1.53**	7.78	7.76	−0.02	*Api5* (apoptosis inhibitor 5)	Prevents apoptosis after growth factor deprivation.
74126	8.08	6.90	**−1.17**	9.56	9.45	−0.11	*Syvn1* (synovial apoptosis inhibitor 1, synoviolin)	Protects cells from ER stress-induced apoptosis
69928	6.41	5.43	**−0.99**	4.58	4.55	−0.03	*Apitd1* (apoptosis-inducing, TAF9-like domain 1)	Cell growth and/or cell death properties in neuroblastoma cells
29810	8.73	8.47	−0.25	8.93	12.59	**3.67**	*Bag3* (BCL2-associated athanogene 3)	Anti-apoptotic activity
237436	nl	nl	-	4.38^nl^	6.31	**1.93**	*Gas2l3* (growth arrest-specific 2 like 3)	Microtubule-actin cross-linking protein
21664	nl	nl	-	11.41	12.95	**1.54**	*Phlda1* (pleckstrin homology-like domain, family A, member 1)	Regulation of apoptosis
17210	7.87	6.97	**−0.90**	9.02	10.06	**1.04**	*Mcl1* (myeloid cell leukemia sequence 1)	Regulation of apoptosis
21929	5.52	5.52	0.00	5.43	6.42	**0.99**	*Tnfaip3* (tumor necrosis factor, alpha-induced protein 3)	Inhibitor of programmed cell death
17873	6.02	6.36	0.34	5.79	6.75	**0.95**	*Gadd45b* (growth arrest and DNA-damage-inducible 45 beta)	Regulation of growth and apoptosis
23882	5.11	5.65	0.54	8.93	7.71	**−1.22**	*Gadd45g* (growth arrest and DNA-damage-inducible 45 gamma)	Regulation of growth and apoptosis

The level of expression in spermatocytes (SC) and hepatocytes (HEP) is given in arbitrary units in logarithmic scale (log2). Changes in gene expression after hyperthermia (at 38°C or 43°C) are shown as SLRs.

*nl* noise level.

During the heat shock response expression of genes coding for some transcriptional regulators involved in the growth, proliferation and differentiation was strongly activated in hepatocytes, but not in spermatocytes (especially *Atf3* and genes coding for components of AP1 transcription factor: JUN and FOS; Table 4). In spermatocytes, a different set of transcriptional regulators was activated (which were not activated in hepatocytes, except for *Zfp36l1*). In spermatocytes numerous transcriptional regulators were repressed, e.g. some components of general transcription factors machinery (Table 4). Some of the transcription factors, whose mRNAs were elevated in hepatocytes during the heat shock response, play a role in the inflammatory responses (e.g. NFKBIZ, NFIL3). Consistently, some other genes involved in the inflammatory and immune responses were significantly up-regulated in hepatocytes (e.g. *Socs3* and genes coding for chemokine ligands; Table 5).

**Table 4:** Selected transcriptional regulators differentially expressed after hyperthermia

**Entrez gene ID**	**Mean SC_C**	**Mean SC_38**	**SLR SC_38 vs C**	**Mean HEP_C**	**Mean HEP_43**	**SLR HEP_43 vs C**	**Gene symbol (full name)**
**A. Induced in spermatocytes, heat shock 38°C**							
224694	4.30^nl^	5.82	**1.52**	nl	nl	-	*Zfp81* (zinc finger protein 81)
16600	8.78	10.28	**1.49**	5.18	5.67	0.49	*Klf4* (Kruppel-like factor 4 (gut)
22768	4.37	5.62	**1.26**	nl	nl	-	*Zfy2* (zinc finger protein 2, Y linked)
73503	9.13	10.34	**1.21**	4.91	5.16	0.25	*Mbd3l1* (methyl-CpG binding domain protein 3-like 1)
208727	5.29	6.49	**1.19**	4.55	4.65	0.09	*Hdac4* (histone deacetylase 4)
54427	6.77	7.95	**1.19**	5.62	5.10	−0.52	*Dnmt3l* (DNA (cytosine-5-)-methyltransferase 3-like)
12224	8.22	9.39	**1.17**	4.91	4.64	−0.27	*Klf5* (Kruppel-like factor 5)
56381	7.18	8.30	**1.12**	6.15	6.04	−0.11	*Spen* (SPEN homolog, transcriptional regulator (Drosophila))
75753	6.08	7.15	**1.07**	5.12	5.42	0.30	*Klf17* (Kruppel-like factor 17)
16870	6.10	7.16	**1.06**	4.96	4.92	−0.04	*Lhx2* (LIM homeobox protein 2)
16601	7.21	8.27	**1.06**	8.39	8.31	−0.08	*Klf9* (Kruppel-like factor 9)
18132	5.99	7.04	**1.05**	nl	nl	-	*Notch4* (Notch gene homolog 4 (Drosophila))
56706	8.61	9.62	**1.01**	8.57	9.17	0.60	*Ccnl1*(cyclin L1)
**B. Repressed in spermatocytes, heat shock 38°C**							
60406	9.25	6.78	**−2.47**	6.10	6.20	0.10	*Sap30* (sin3 associated polypeptide)
74197	8.42	6.14	**−2.28**	6.83	7.04	0.21	*Gtf2e1* (general transcription factor II E, polypeptide 1 (alpha subunit))
209707	7.30	5.26	**−2.04**	4.54	4.85	0.31	*Lcorl* (ligand dependent nuclear receptor corepressor-like)
15904	8.88	6.88	**−2.00**	4.79	4.84	0.05	*Id4* (inhibitor of DNA binding 4)
71041	9.77	7.93	**−1.83**	6.81	7.13	0.32	*Pcgf6* (polycomb group ring finger 6)
26919	8.66	6.89	**−1.77**	6.16	6.55	0.40	*Zfp346* (zinc finger protein 346)
319944	6.84	5.12	**−1.73**	5.53	5.44	−0.09	*Taf2* (TAF2 RNA polymerase II, TATA box binding protein (TBP)-associated factor)
22702	6.88	5.22	**−1.66**	nl	nl	-	*Zfp42* (zinc finger protein 428)
231329	8.77	7.34	**−1.43**	9.50	9.33	−0.17	*Polr2b* (polymerase (RNA) II (DNA directed) polypeptide B)
102334	7.95	6.48	**−1.47**	5.90	6.18	0.28	*Ankrd10* (ankyrin repeat domain 10)
78912	6.92	5.55	**−1.38**	5.94	5.88	−0.06	*Sp2* (Sp2 transcription factor)
19009	7.02	5.69	**−1.33**	4.81	4.79	−0.01	*Pou6f1* (POU domain, class 6, transcription factor 1)
20687	4.86	3.58	**−1.29**	7.14	7.07	−0.08	*Sp3* (trans-acting transcription factor 3)
75507	9.42	8.19	**−1.22**	8.05	7.90	−0.15	*Pou5f2* (POU domain class 5, transcription factor 2)
100683	7.50	6.29	**−1.21**	6.94	6.84	−0.10	*Trrap* (transformation/transcription domain-associated protein)
22632	8.75	7.60	**−1.15**	9.01	9.33	0.32	*Yy1* (YY1 transcription factor)
16476	5.86	4.75	**−1.11**	8.41	12.47	**4.07**	*Jun* (Jun oncogene)
14581	5.65	4.54	**−1.11**	nl	nl	-	*Gfi1*(growth factor independent 1)
23894	7.29	6.20	**−1.09**	6.27	6.25	−0.02	*Gtf2h2* (general transcription factor II H, polypeptide 2)
**C. Induced in hepatocytes, heat shock 43°C**							
11910	6.38	5.87	−0.51	6.44	10.80	**4.36**	*Atf3* (activating transcription factor 3)
16476	5.86	4.75	**−1.11**	8.41	12.47	**4.07**	*Jun* (Jun oncogene)
23849	nl	nl	-	4.63	7.55	**2.92**	*Klf6* (Kruppel-like factor 6)
14281	7.52	6.81	−0.71	9.38	11.94	**2.56**	*Fos* (FBJ osteosarcoma oncogene)
13653	7.50	7.65	0.15	10.66	13.03	**2.38**	*Egr1* (early growth response 1)
215418	7.41	7.10	−0.31	7.23	9.28	**2.05**	*Csrnp1* (cysteine-serine-rich nuclear protein 1)
80859	5.12	4.92	−0.20	6.08	7.91	**1.83**	*Nfkbiz* (nuclear factor of kappa light polypeptide gene enhancer in B-cells inhibitor, zeta)
18030	nl	nl	-	6.28	7.99	**1.71**	*Nfil3* (nuclear factor, interleukin 3, regulated)
15370	9.23	9.66	0.43	8.51	10.21	**1.69**	*Nr4a1* (nuclear receptor subfamily 4, group A, member 1)
16477	5.86	5.44	−0.42	8.31	9.90	**1.59**	*Junb* (Jun-B oncogene)
12053	4.86	5.17	0.31	5.74	7.05	**1.31**	*Bcl6* (B-cell leukemia/lymphoma 6)
15982	6.92	7.31	0.40	7.22	8.41	**1.20**	*Ifrd1* (interferon-related developmental regulator 1)
12192	6.09	7.03	**0.94**	10.05	11.26	**1.21**	*Zfp36l1* (zinc finger protein 36, C3H type-like 1)
13654	nl	nl	-	4.09^nl^	5.28	**1.19**	*Egr2* (early growth response 2)
17133	4.93	5.32	0.39	5.94	7.04	**1.10**	*Maff* (v-maf musculoaponeurotic fibrosarcoma oncogene family, protein F (avian))
271377	7.42	7.30	−0.13	7.42	8.49	**1.07**	*Zbtb11* (zinc finger and BTB domain containing 11)
81703	4.59	4.59	0.00	5.07	6.11	**1.04**	*Jdp2* (Jun dimerization protein 2)
**D. Repressed in hepatocytes, heat shock 43°C**							
21412	nl	nl	-	5.82	4.75	**−1.08**	*Tcf21* (transcription factor 21

The level of expression in spermatocytes (SC) and hepatocytes (HEP) is given in arbitrary units in logarithmic scale (log2). Changes in gene expression (at 38°C or 43°C) are shown as SLRs.

*nl* noise level.

**Table 5:** The most significant changes in expression of genes involved in the inflammatory and immune responses

**Entrez gene ID**	**Mean SC_C**	**Mean SC_38**	**SLR SC_38 vs C**	**Mean HEP_C**	**Mean HEP_43**	**SLR HEP_43 vs C**	**Gene symbol (full name)**
16071	4.07^nl^	5.30	**1.23**	7.09	7.49	0.40	*Igkc* (immunoglobulin kappa constant)
11450	7.57	8.78	**1.21**	4.85	4.90	0.05	*Adipoq* (adiponectin, C1Q and collagen domain containing)
142687	5.02	6.12	**1.10**	nl	nl	-	*Asb14* (ankyrin repeat and SOCS box-containing 14)
58185	5.42	6.49	**1.07**	5.89	5.78	−0.11	*Rsad2* (radical S-adenosyl methionine domain containing 2)
71785	4.36^nl^	5.38	**1.02**	nl	nl	-	*Pdgfd* (platelet-derived growth factor, D polypeptide)
217845	5.03	6.04	**1.02**	4.78	4.89	0.11	*Ifi27l2b* (interferon, alpha-inducible protein 27 like 2B)
57444	8.56	9.57	**1.01**	5.36	5.19	−0.17	*Isg20* (interferon-stimulated protein)
54131	8.33	7.20	**−1.13**	8.51	8.53	0.02	*Irf3* (interferon regulatory factor 3)
67781	7.26	6.25	**−1.02**	5.93	5.80	−0.13	*Ilf2* (interleukin enhancer binding factor 2)
12702	5.36	5.32	−0.04	6.55	9.88	**3.32**	*Socs3* (suppressor of cytokine signaling 3)
20310	5.48	5.75	0.27	4.57	6.64	**2.06**	*Cxcl2* (chemokine (C-X-C motif) ligand 2)
12475	6.19	6.17	−0.02	7.20	9.03	**1.82**	*Cd14* (CD14 antigen)
12522	4.52	4.50	−0.02	4.76	6.50	**1.73**	*Cd83* (CD83 antigen)
20210	6.92	6.83	−0.10	7.97	9.56	**1.59**	*Saa3* (serum amyloid A 3)
14825	6.13	6.49	0.36	8.53	10.11	**1.57**	*Cxcl1* (chemokine (C-X-C motif) ligand 1)
21824	4.68	4.70	0.02	5.12	6.64	**1.52**	*Thbd* (thrombomodulin)
211770	5.29	5.63	0.35	6.47	7.99	**1.52**	*Trib1* (tribbles homolog 1 (Drosophila))
16175	5.18	5.62	0.44	6.26	7.55	**1.28**	*Il1a* (interleukin 1 alpha)
11504	4.60	4.82	0.22	5.71	6.80	**1.09**	*Adamts1* (a disintegrin-like and metallopeptidase (reprolysin type) with thrombospondin type 1 motif, 1)
278180	4.49^nl^	4.62	0.13	9.28	7.69	**−1.59**	*Vsig4* (V-set and immunoglobulin domain containing 4)
110454	6.40	6.74	0.34	9.50	8.36	**−1.14**	*Ly6a* (lymphocyte antigen 6 complex, locus A)

The level of expression in spermatocytes (SC) and hepatocytes (HEP) is given in arbitrary units in logarithmic scale (log2). Changes in gene expression after hyperthermia (at 38°C or 43°C) are shown as SLRs.

*nl* noise level.

Data obtained from microarrays analyses were further verified and visualized by semi-quantitative RT-PCR on independent material. Expression of selected genes was analyzed in isolated spermatocytes, and in whole testes and liver, after 2 h, 4 h, 6 h and 24 h of recovery from heat shock. These analyses revealed that the level of transcripts strongly elevated after hyperthermia at 43°C in somatic cells (both in liver and testes) returned to the steady-state level 24 h after treatment (Figure 2 and Additional file 9: Figure S1 [24]). Analyses confirmed also a lack of activation of *Hsp* genes (and other genes strongly activated in hepatocytes) in isolated spermatocytes up to 24 h after treatment. RT-PCR analyses generally confirmed data obtained from microarrays studies.

### Differences in HSF1 binding to DNA between somatic and spermatogenic cells

**Figure 3:**
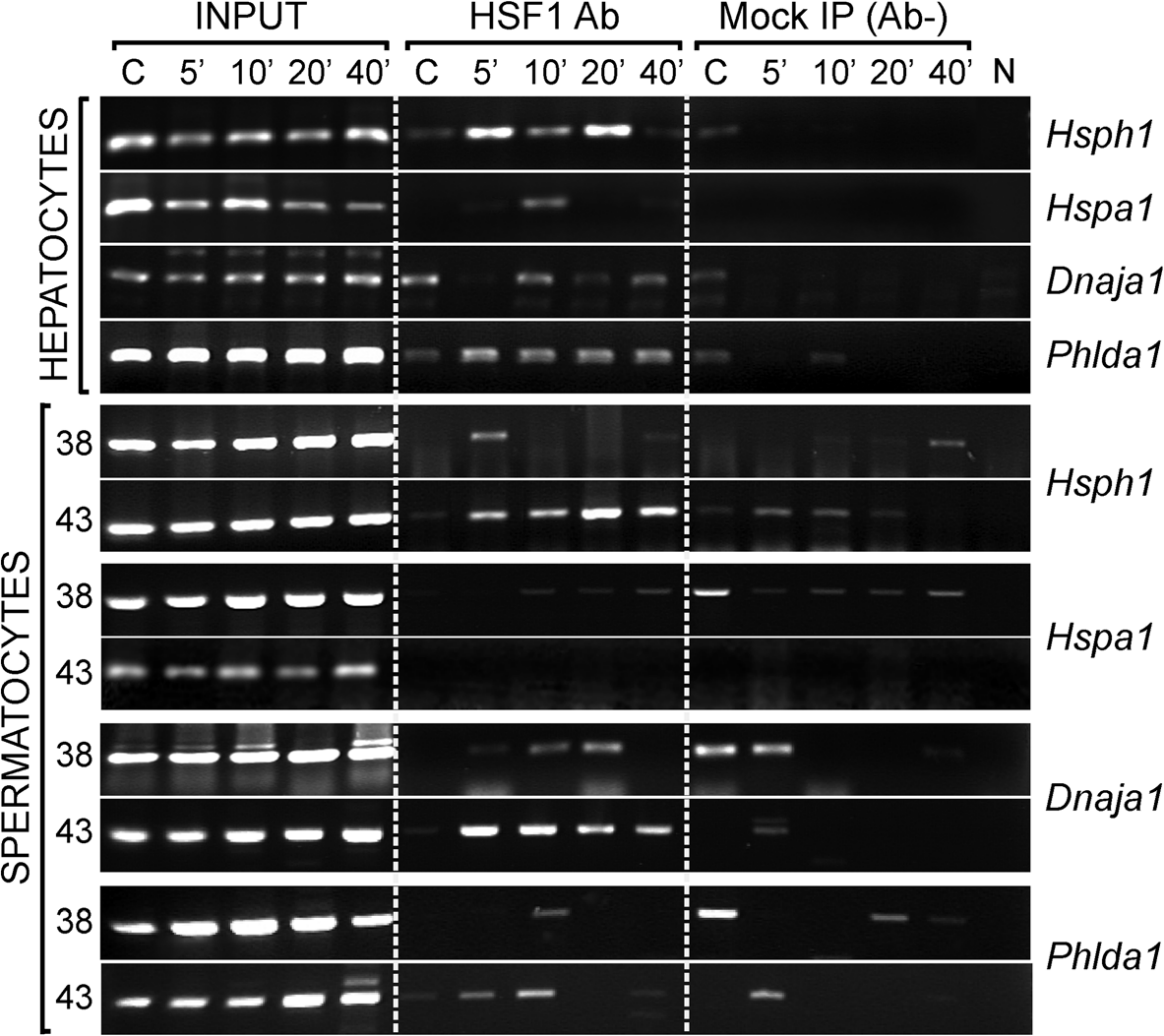
**Kinetics of HSF1 activation.** ChIP analysis of HSF1 binding to promoters of selected genes in hepatocytes and spermatocytes subjected to heat shock in different conditions (from 5 to 40 minutes, and temperatures 38°C or 43°C; hepatocytes were heat shocked only at 43°C). N, PCR-negative control without template.

**Figure 4:**
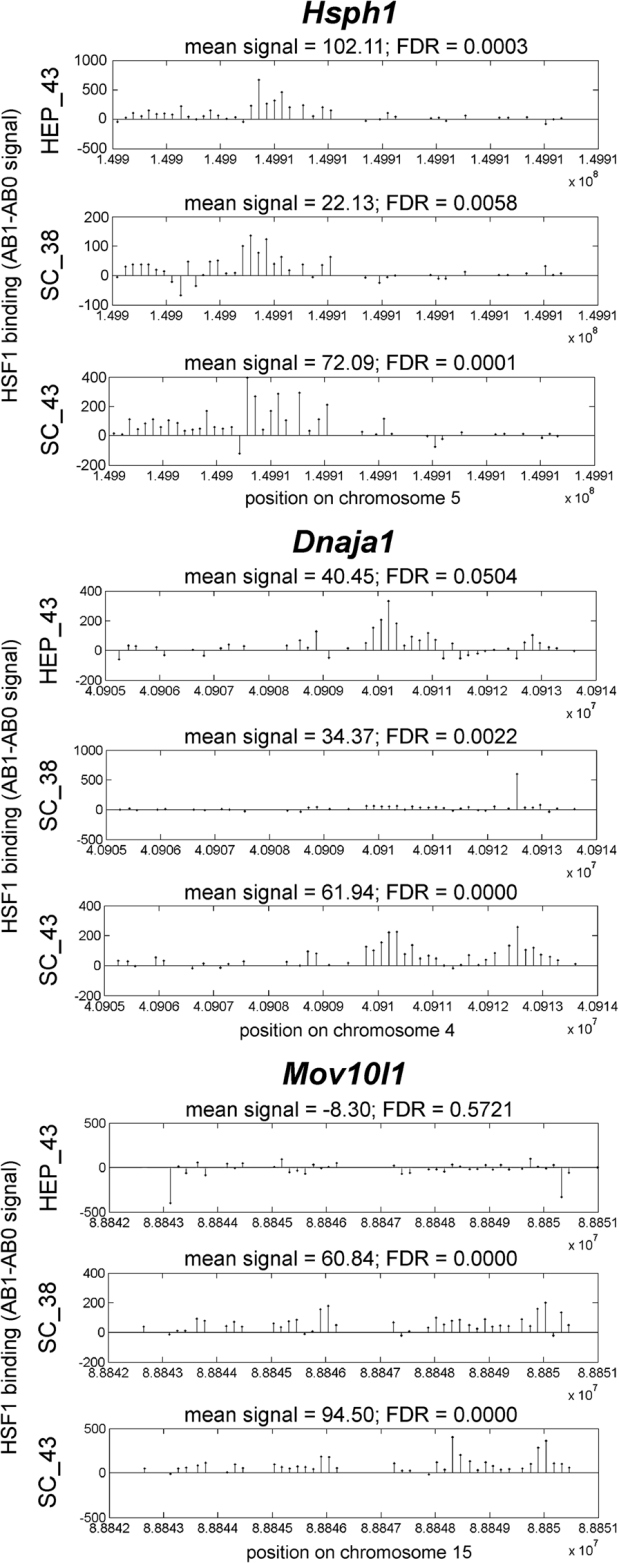
**Graphical representation of HSF1 binding to the promoters of *Hsph1*, *Dnaja1 *and *Mov10l1 *genes following hyperthermia in hepatocytes and spermatocytes.** Results of ChIP on chip analysis are shown. Each line represents the signal from one probeset and is shown with reference to the position on the chromosome (numbered according to NCBIv36 of the mouse genome).

HSF1 is activated at elevated temperatures very rapidly, and the strongest HSF1 binding to DNA was observed after 2–20 minutes of heat shock treatment, depending on experimental model [27]. To find out the optimal conditions of HSF1 binding in our experimental system we first analyzed by ChIP-PCR the ability of HSF1 to bind to promoters of *Hsph1*, *Hspa1*, *Dnaja1* and *Phlda1* (genes already known to be regulated by HSF1). Binding of HSF1 was analyzed after 5, 10, 20 and 40 minutes of incubation of cells at elevated temperatures. Isolated hepatocytes were incubated at 43°C. Isolated spermatocytes were incubated at 38°C or 43°C. Observed HSF1 binding varied depending on the gene and conditions of heat shock (time and temperature; Figure 3). In spermatocytes during heat shock at 38°C the HSF1 binding was generally much weaker than at 43°C (both in spermatocytes and hepatocytes), and a prolonged period of hyperthermia (40 min) did not straighten HSF1 binding. Interestingly, in the case of *Hspa1* genes, the most activated genes during heat shock in somatic cells, we observed only moderate HSF1 binding to the promoters in hepatocytes (and no binding in spermatocytes). In further in-depth ChIP on chip studies our goal was not study of the kinetics of HSF1 binding but finding the broadest spectrum of its targets. Thus chromatin immunoprecipitation was done on cells heat shocked for 5, 10 and 20 minutes, than combined in one sample. ChIP on chip analyses were performed on Affymetrix mouse promoter tiling arrays to find out the molecular targets of HSF1 in all promoter regions. Untreated cells (control) and cells subjected to hyperthermia (hepatocytes at 43°C and spermatocytes at 38°C or 43°C) were analyzed, and the mean signals (in log2 scale) for each probeset on the array have been calculated in samples with antibody (denoted as AB1) and in samples without antibody (denoted as AB0). Binding of HSF1 was assessed as AB1-AB0 value. An example of analysis showing the graphical representation of AB1-AB0 signals from each probeset distributed on promoters is shown in Figure 4. Finally, numerical value estimating HSF1 binding was calculated in arbitrary units for each promoter (mean signal from all probesets annotated to the promoter). Results of such analyzes obtained for each promoter are presented in Additional file 10[24] (containing additional information about expression level of the corresponding transcript).

**Figure 5:**
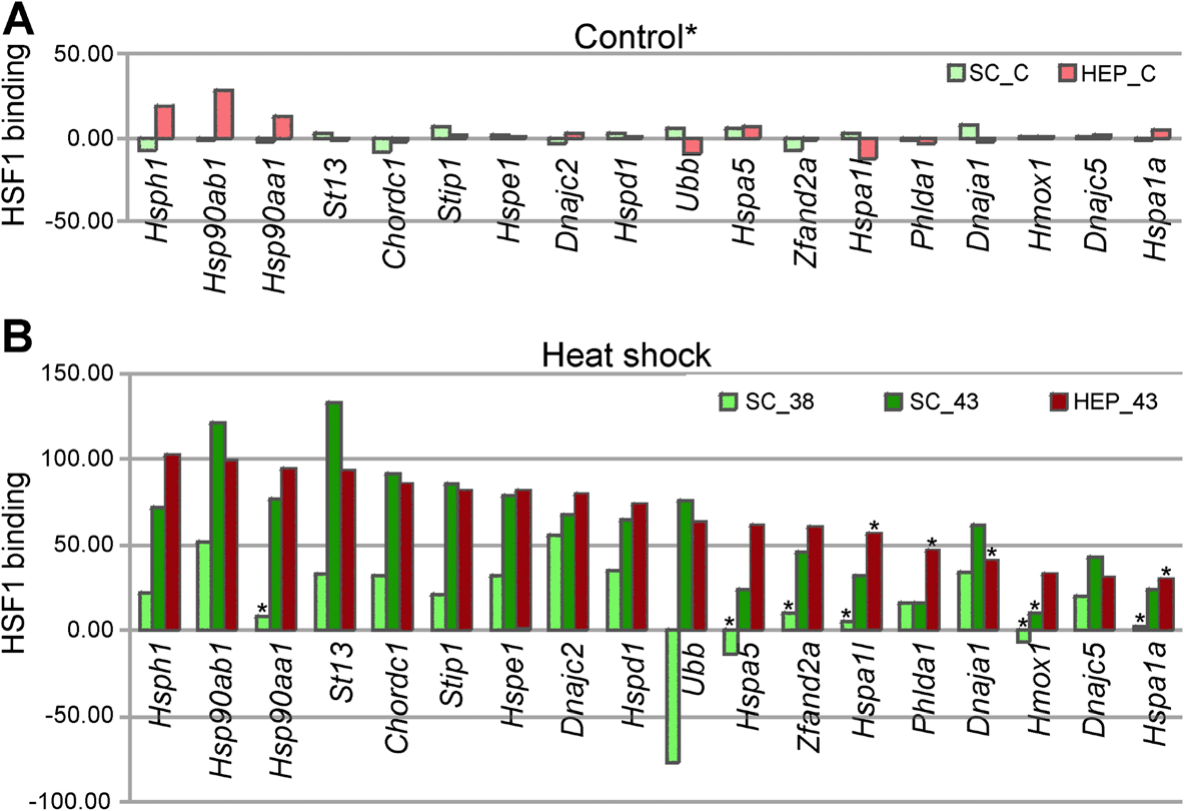
**Binding of HSF1 estimated in ChIP on chip analysis to promoters of selected genes. (A)** Binding at physiological and **(B)** elevated temperatures. HSF1 binding is calculated as AB1-AB0 mean signal in arbitrary units. *FDR > 0.05.

**Figure 6:**
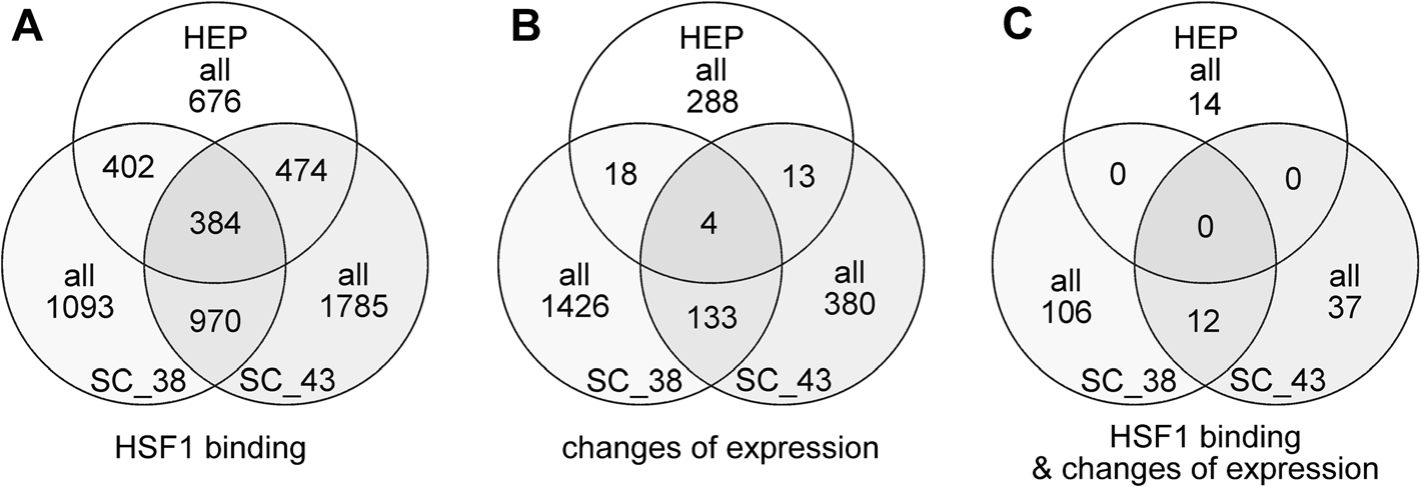
**Overlap of genes affected by hyperthermia in hepatocytes and spermatocytes.** Numbers of genes showing binding of HSF1 **(A)**, modulated expression **(B)**, and both **(C)**. For HSF1 binding values AB1-AB0 ≥ 15 and FDR < 0.05 were taken into consideration; in the case of changes in expression level – range of SLR presented in Table 1.

Searching for HSF1 binding at the normal physiological temperature in spermatocytes we found only a few genes with statistically significant signals (false discovery rate, FDR < 0.05), while in hepatocytes there was no such genes (Table 6). ChIP on chip analyzes revealed that following hyperthermia promoters of several *Hsp* genes and some other genes already known to be regulated by HSF1 [28-30], interact with HSF1 in both hepatocytes and spermatocytes (Figure 5). In this group of genes, binding of HSF1 at the physiological temperature was detected only to the *Hsph1* and *Hsp90ab1* promoters, although the significance of the signal was only marginal (FDR > 0.05). In general, heat shock strongly induced HSF1 binding (yet binding at 38°C in spermatocytes was usually weaker). Interestingly, HSF1 interacted with more genes in spermatocytes than in hepatocytes, although in hepatocytes the binding was stronger (Table 6). More than 50% of genes interacting with HSF1 in hepatocytes, were bound by HSF1 also in spermatocytes (Figure 6A). Interestingly, in hepatocytes (but not in spermatocytes) the uppermost HSF1 binding following heat shock was observed for sequences located on the Chromosome Y (Additional file 11: Table S5 [24]). Genes showing the uppermost HSF1 binding in spermatocytes also showed strong binding in hepatocytes (Additional file 11: Table S5 [24]). The *Gene to GO BP* test showed that genes bound by HSF1 in both hepatocytes and spermatocytes have the strongest representation in following classes: RNA processing and transport, DNA replication, repair, recombination and chromatin modification, protein folding, translation and ubiquitination, Golgi organization, zinc ion transport and microtubule cytoskeleton. As could be expected, HSF1-bound genes involved in spermatogenesis, meiosis and mitosis (and related to cell cycle and cell division) have much higher representation in spermatocytes than in hepatocytes (Additional file 12[24]).

**Table 6:** Statistics of HSF1 binding

	**Spermatocytes**			**Hepatocytes**	
	**Control**	**HS_38°C**	**HS_43°C**	**Control**	**HS_43°C**
Number of genes	4	1093	1785	0	676
Mean HSF1 binding signal ± SD	23.8 ± 6.6	31.4 ± 20.5	33.1 ± 19.7	-	54.1 ± 65.3

Only values AB1-AB0 ≥ 15 with FDR < 0.05 are included. *HS* heat shock, *SD* standard deviation.

### Correlation between HSF1 binding and modulation of gene expression during heat shock

In the next step of analysis, we selected genes that both were bound by HSF1 and whose expression was changed in cells subjected to hyperthermia. We found significant changes of expression only in a fraction of genes that interacted with HSF1 (Figure 6C). In general, the binding of HSF1 was correlated with an activation of the transcription in hepatocytes and with a repression in spermatocytes (Table 7). Top HSF1-bound genes induced or repressed in spermatocytes and hepatocytes following heat shock are shown in Table 8 (all results are presented in Additional file 13[24]). In the selected genes HSF1 binding was usually observed both in hepatocytes and in spermatocytes. Nevertheless, similar HSF1 binding did not result in similar changes in expression when both types of cells were compared. Even in spermatocytes subjected to hyperthermia at 38°C and 43°C, HSF1-bound genes were differentially regulated after heat shock. This finding suggests that factors responsible for HSF1 activation are not only cell-type specific, but also dependent on the temperature. We also searched for a general statistical correlation between hyperthermia-induced changes in gene expression and HSF1 binding. All genes where in case of expression SLR ≠ 0 and in case of HSF1 binding AB1 > AB0 were included into analysis; results are presented in Additional file 14: Table S6 [24]. The correlation was found between expression and actual HSF1 binding at elevated temperatures but not at physiological temperature. It is noteworthy that a negative statistical correlation was observed for both up- and down-regulated genes in spermatocytes, i.e. enhanced HSF1 binding was associated with a decreased expression. In contrast, a positive statistical correlation was observed for up-regulated genes in hepatocytes, i.e. enhanced HSF1 binding was associated with an increased expression.

**Table 7:** Correlation of HSF1 binding following heat shock and subsequent changes in the transcription level

	**Binding of HSF1**					
	**Induction**			**Repression**		
	**Number of genes**	**% of all induced**	**% of all bound**	**Number of genes**	**% of all repressed**	**% of all bound**
Spermatocytes HS_38°C	2	0.39%	0.18%	104	11.44%	9.52%
Spermatocytes HS_43°C	1	5.56%	0.06%	36	9.94%	2.02%
Hepatocytes HS_43°C	11	7.10%	1.63%	3	2.26%	0.44%

For HSF1 binding values AB1-AB0 ≥ 15 and FDR < 0.05 were taken into consideration; in case of changes in expression level – range of SLRs presented in Table 1.

**Table 8:** Top HSF1-bound genes induced or repressed in spermatocytes and hepatocytes following heat shock

**Gene symbol**	**Mean expression**			**SLR**		**Mean expression**		**SLR HEP_43 vs C**	**HSF1 binding (AB1-AB0)**		
	**SC_C**	**SC_38**	**SC_43**	**SC_38 vs C**	**SC_43 vs C**	**HEP_C**	**HEP_43**		**SC_38**	**SC_43**	**HEP_43**
**A. Induced in spermatocytes, heat shock 38°C**											
*Spen*	7.18	8.30	7.06	**1.12**	−0.12	6.35	6.04	−0.31	**14.05**	50.30	12.89*
*Asb14*	5.02	6.12	5.05	**1.10**	0.03	nl	nl	-	**24.00**	20.80	-
*Adam21*	8.02	9.05	8.01	**1.03**	−0.01	5.65	5.98	0.34	**21.03**	17.76*	-
*1700109H08Rik*	9.46	10.41	9.42	**0.95**	−0.04	4.71	4.85	0.14	**28.80**	49.60	35.0
**B. Repressed in spermatocytes, heat shock 38°C**											
*6330503K22Rik*	10.09	7.93	10.11	**−2.16**	0.03	5.09	5.65	0.56	**65.99**	82.04	27.33
*Topbp1*	8.01	6.44	7.47	**−1.57**	−0.54	5.68	5.80	0.11	**122.90**	96.50	160.08*
*Fam178a*	7.88	6.36	7.65	**−1.52**	−0.23	7.13	7.48	0.35	**67.15**	76.91	66.54
*Specc1l*	8.78	7.47	8.57	**−1.31**	−0.22	7.38	7.30	−0.08	**82.75**	113.69	46.78
*Cdc42*	7.79	6.51	7.29	**−1.29**	−0.50	10.04	9.84	−0.20	**38.25**	35.53	28.38
*Rbm27*	7.33	6.09	7.26	**−1.24**	−0.07	7.08	7.11	0.03	**42.30**	54.71	31.60
*Trrap*	7.50	6.29	7.18	**−1.21**	−0.32	6.94	6.84	−0.10	**54.75**	78.92	85.63
*Piwil2*	9.36	8.16	9.14	**−1.20**	−0.22	4.57	4.53	−0.04	**58.29**	69.75	-
*Prpf4b*	7.43	6.32	7.39	**−1.11**	−0.04	7.32	7.57	0.25	**63.92**	60.88	47.68
*Dock7*	8.23	7.13	7.95	**−1.10**	−0.29	6.73	6.74	0.01	**53.87**	80.68	58.32
*Mov10l1*	8.54	7.47	8.22	**−1.07**	−0.32	5.80	6.03	0.23	**60.84**	94.50	-
*BC026590*	7.64	6.60	7.21	**−1.04**	−0.42	5.08	5.19	0.11	**40.27**	40.98	51.39
*Srp68*	9.74	8.72	9.61	**−1.01**	−0.12	8.89	8.93	0.04	**38.09**	61.12	43.79
**C. Induced in hepatocytes, heat shock 43°C**											
*Hsph1*	7.77	7.64	7.84	−0.13	0.07	9.18	11.65	**2.47**	22.13	72.09	**102.11**
*Zfand2a*	6.57	6.20	6.56	−0.37	−0.01	6.43	8.44	**2.01**	10.50	46.06	**60.47**
*Phlda1*	nl	nl	nl	-	-	11.41	12.95	**1.54**	16.38	15.60	**47.09**
*Mpp4*	4.57	4.55	4.80	−0.02	0.24	4.89	6.04	**1.15**	28.00	51.81	**47.75**
*Hsp90ab1*	9.94	9.35	9.89	−0.59	−0.05	11.29	12.31	**1.02**	51.71	120.95	**99.84**
*Slc10a2*	nl	nl	nl	-	-	4.08^nl^	5.10	**1.02**	-	-	**54.11**
*Hspe1*	7.03	7.09	6.91	0.06	−0.12	8.63	9.64	**1.01**	31.92	78.72	**81.14**
*Prdm15*	5.05	4.94	4.83	−0.11	−0.23	4.70	5.49	**0.79**	27.64	47.05	**42.34**
*Rbbp6*	6.53	6.23	6.49	−0.30	−0.04	5.76	6.51	**0.75**	51.16	61.71	**93.60**
**D. Repressed in hepatocytes, heat shock 43°C**											
*Cyp4a10*	nl	nl	nl	-	-	8,25^**#**^	4,63	**−3,62**	-	-	**31,90**
*Sdc4*	5.96	5.94	5.92	−0.03	−0.05	11.47^**#**^	9.90^**#**^	**−1.57**	-	-	**25.93**
*Pabpc2*	12.54	12.23	12.30	−0.31	−0.24	5.42	4.67	**−0.75**	-	19.81*	**17.61**

*FDR > 0.125; *nl* noise level, ^**#**^high standard deviation (in a range 1.1 – 3.6).

Finally, we searched for HSF1-regulated genes putatively involved in cell type- specific response to stress conditions. Among genes that bind HSF1 and are activated in spermatocytes treated at 38°C but not in heat shocked hepatocytes the most interesting seems to be *Spen* (SPEN homolog, transcriptional regulator (Drosophila)), which is a transcriptional repressor. Among genes that bind HSF1 and are negatively regulated in spermatocytes the biggest group are those involved in DNA replication and cell division, regulation of transcription and RNA processing, protein folding and degradation, and intracellular transport (listed and characterized in Additional file 15: Table S7 [24]). Interestingly, several genes that are bound by HSF1 and are negatively regulated in spermatocytes treated with hyperthermia at 38°C are essential for spermatogenesis (Table 9). It has been shown that their knock-out leads to defects in spermatogenesis. The most interesting in this group are genes involved in the repression of transposable elements: *Tdrd1*, *Piwil2* and *Mov10l1*. We verified expression of some of these genes using quantitative RT-PCR analysis on independent material and confirmed significant down-regulation of *Tdrd1* and *Mov10l1* (Additional file 16: Figure S2 [24]).

**Table 9:** Characteristics of genes necessary for spermatogenesis whose repression following heat shock in spermatocytes correlates with HSF1 binding

**Gene symbol (full name)**	**Changes in expression (SLR)**			**HSF1 binding (AB1-AB0)**			**Function**
	**SC_38 vs C**	**SC_43 vs C**	**HEP_43 vs C**	**SC_38**	**SC_43**	**HEP_43**	
*Dnaja1* (DnaJ (Hsp40) homolog, subfamily A, member 1)	−1.09	−0.07	1.17	34.4	69.9	40.5	Cochaperone of HSP70s in protein folding and mitochondrial protein import. Its loss in mice led to severe defects in spermatogenesis [31].
*Celf1* (CUGBP, Elav-like family member 1)	−1.07	−0.01	0.19	35.1	48.3	24.5	Post-transcriptional regulation: pre-mRNA alternative splicing, mRNA translation and stability. Required for completion of spermatogenesis [32].
*Spo11* (sporulation protein, meiosis-specific, SPO11 homolog (S. cerevisiae))	−1.19	0.07	−0.03	33.0	53.3	-	A type II like topoisomerase; required for meiotic recombination [33,34].
*Piwil2* (piwi-like homolog 2 (Drosophila))	−1.20	−0.22	−0.04	58.3	69.8	-	Participates in the repression of transposable elements in spermatogenic cells; involved in translation regulation [35,36].
*Tdrd1* (tudor domain containing 1)	−1.57	−0.40	0.42	23.8	48.1	-	Participates in the repression of transposable elements in spermatogenic cells [37].
*Mov10l1* (Moloney leukemia virus 10-like 1, homolog (mouse))	−1.07	−0.32	0.23	60.8	94.5	-	Putative RNA helicase, essential for silencing retrotransposons in the mouse male germline [38,39].

## Discussion

The changes in the global gene expression profiles following heat shock were already studied in different organisms (from bacteria to mammals), and in different types of cells or tissues [8,40-46], even in mouse liver [45] and testes [47-49]. Although some differences in heat shock response between somatic cells exist, in all of them pro-survival signaling pathways are activated. Their activation was also observed in mouse testes when changes in whole tissue were analyzed [47,49]. The strategy used in the present study allowed us to find differences in the heat shock response between somatic and spermatogenic germ cells. By isolating the spermatogenic cells we avoided the overlapping of signals from different types of cells. We found that genes selected by Li et al. [49] as activated in whole testes during heat shock at 43°C (e.g. *Hspa1a*, *Egr1*, *Fos*, *Socs3*, *Hspa8*, *Jun* and *Dnaja4*) are not induced in spermatogenic cells, although they are strongly induced in hepatocytes. In fact, the lack of strong activation of *Hsp* genes in isolated spermatogenic cells documented in the present study is consistent with some previous findings [15,16]. Hence, our data confirms basic differences between spermatogenic cells and somatic cells (exemplified by hepatocytes) in the transcriptional response to stress.

Spermatogenic cells do not activate “universal” pro-survival pathways in response to stress. However, because a large fraction of genes affected in spermatocytes during hyperthermia remains uncharacterized, identification of the signaling pathways involved in stress response is difficult. Additionally, considerably more genes are repressed than activated, and primarily pathways connected with RNA processing are strongly repressed under stress conditions. Posttranscriptional regulation is of critical importance during mammalian spermatogenesis. The testis has the greatest enrichment of tissue-specific splicing [50]. Also alternative promoters and polyA sites, antisense transcripts, necessity of the mRNA storage for a longer time before translation, and a different class of regulatory RNAs contribute to transcriptome diversification in testis [51]. Thus, deregulated RNA processing during heat shock could result in serious disturbances of spermatogenesis.

HSF1 binding to DNA was studied on a genome-wide scale in Saccharomyces and Drosophila cells or embryos [6,7,52,53], as well as in mammalian cells [8,41,54], including mouse testis at a physiological temperature [55]. All these studies suggest that HSF1 may regulate diverse cellular processes that extend far beyond protein folding, the general role of HSPs that are the major targets of this factor. Here we found that several genes involved in processes other than protein folding (mainly in RNA processing and DNA replication, recombination and repair) were bound by HSF1, and that many genes were similarly bound by HSF1 in both hepatocytes and spermatocytes despite a completely different nature in the resulting transcriptional response. Recently, it has also been noted that HSF1 has important cell-specific functions at a normal physiological temperature. It regulates cancer-specific genes that support oncogenic processes in highly tumorigenic cells, in which it is more active than in less malignant cells [54]. In testis under physiologically normal conditions, HSF1 is required for transcriptional regulation of sex chromosomal multicopy genes [55]. Here we have observed that only a few genes were strongly bound by HSF1 in purified spermatocytes not subjected to hyperthermia.

Although HSF1 binding in hepatocytes and spermatocytes shows a relatively similar pattern, transcription of HSF1-bound genes is regulated in an apparently different way in these cells. Additionally, in spermatocytes heat shocked at 38°C and 43°C the changes in transcription are markedly different despite a rather similar pattern of HSF1 binding. Even though HSF1 binds more effectively to DNA in cells treated at 43°C, changes in the transcription level of HSF1-bound genes are much stronger in spermatocytes treated at 38°C. We have concluded that regulation of transcription by HSF1 binding is not only a cell type-specific mechanism, but it is also affected by temperature. HSF1 binding to the DNA in most cases does not correlate with significant changes in gene expression (on the other hand, HSF1 controls only a subset of the genes altered by heat shock). It is not surprising because the DNA-binding ability and the transactivation competence of HSF1 are regulated independently. HSF1 activity is modulated not only by interactions with chaperones but also by numerous posttranslational modifications including phosphorylation, acetylation, and sumoylation [56]. Additionally, independently of its transactivating activities, HSF1 can control the chromatin organization in response to heat shock [57]. HSF1 mediates a genome-wide and massive histone deacetylation interacting with HDAC1 and HDAC2. Moreover, the transcriptional response to stress may be regulated by microRNAs. Recently, Wilmink et al. [58] identified a group of miRNAs (mostly not annotated) differentially expressed following hyperthermia in dermal fibroblasts. One should not exclude the cell-type specificity of miRNA-related mechanisms in the regulation of heat shock response, which introduces an additional level of complexity in its regulation. In line with this possibility, expression of an extra class of small RNAs molecules involved in gene silencing, so called piRNA, is expressed specifically in spermatogenic cells [59].

The most unexpected finding of our study is that binding of HSF1 could result in inhibition of the transcription on a wide scale. HSF1 is well known as a transcriptional activator, and only a few reports have shown that HSF1 can act as a transcriptional repressor of specific genes involved in the acute phase response and inflammation (like *Il1b*, *Tnf*, *Fos* or *Csf1*; [60,61]. However, in that case HSF1 antagonizes the activity of other transcription factors (by direct protein-protein interactions) and blocks the activation of the genes mentioned above, after stimulation (e.g. by lipopolysaccharide). Recently, it was suggested that HSF1 can inhibit transcription on a wider scale by interactions with Alu elements containing HSF binding sites [62]. Such sequences are frequently present in the corresponding genomic regions of the down-regulated transcripts in antisense orientation. Therefore, the antisense-mediated mechanism of inhibition of the transcription following heat shock was proposed. This study additionally implies that the direction of regulation (positive versus negative) is influenced by the location of the HSF1-binding site, what was also suggested by Mendillo et al. [54]. They stated that genes positively regulated by HSF1 were more likely bound at the promoter, whereas negatively regulated were more frequently bound in distal regions. Promoter tiling microarrays used in our study limited analyzes to promoter regions. Hence, it cannot be excluded that analyzing HSF1 binding by ChIP-seq could enable the identification of even more genes repressed following HSF1 binding. Furthermore, other transcription factors (that are differentially expressed in spermatocytes and hepatocytes) could interfere with changes in gene expression following hyperthermia, in either HSF1-dependent or independent mechanisms.

Among HSF1-bound genes negatively regulated in spermatocytes in response to hyperthermia, the biggest group is involved in DNA replication and cell division. They encode for proteins required for centrosome duplication, meiotic recombination and spindle organization, among others. Thus, disturbances of their expression can directly influence the progression of meiosis. Other HSF1-bound genes specifically down-regulated in spermatocytes are involved in RNA processing (synthesis, splicing and transport) and transcriptional regulation, which also may have a huge impact on spermatogenesis. HSF1-dependent pathways of protein folding and degradation were also differentially expressed in spermatocytes and hepatocytes. The greatest difference was noted in the case of *Dnaja1* expression. DNAJA1 is generally a widely expressed co-chaperone that together with multiple HSP70 proteins takes part in protein folding [63]. In *Dnaja1* null mice, defects in fertility were observed. However, the defects resulted from a lack of DNAJA1 in Sertoli cells rather than in spermatogenic cells. Although a lack of DNAJA1 in Sertoli cells leads to aberrant androgen signaling, disruption of Sertoli–germ cell contact and severe defects in spermiogenesis [31], it is not known what is a consequence of its lack in spermatogenic cells.

Functionally essential for spermatogenesis and male fertility are some other HSF1-bound genes negatively regulated in spermatocytes following heat shock (listed in Table 9). Arrest of spermiogenesis and increased apoptosis was observed in testes from *Celf1* knock-out males [32]. CELF1 regulates the alternative splicing, which is of a greater importance to spermatogenic cells than to other cells. However it is broadly expressed and belongs to a bigger family of RNA binding proteins, that could possibly perform overlapping functions. Thus, is seems that heat shock-induced down-regulation of *Celf1* (as well as *Dnaja1*) could be important, but not critical for induction of apoptosis in spermatocytes.

Disruption of spermatogenesis before the end of meiotic division was observed in *Spo11*, *Piwil2*, *Tdrd1* and *Mov10l1* knockout mice. All these genes, which we report to be repressed in HSF1-dependent manner, are expressed at a high level and almost exclusively in testes (according to BioGPS database; [64]). SPO11 is a type II like topoisomerase that generates double-strand breaks during meiosis. Consequently, mouse null *Spo11* spermatocytes fail to synapse chromosomes and progress beyond the zygotene stage of meiosis [33,34]. PIWIL2, TDRD1 and MOV10L1 are all involved in mobile elements silencing, which is carried out in the primordial mouse testis. Lack of these proteins results in de-repression of retrotransposons (mainly LINE1) [38,65,66]. PIWIL2 (MILI) belongs to the PIWI (P-element-induced wimpy testis) proteins family which also contains PIWIL1 (MIWI) and PIWIL4 (MIWI2). Following heat shock in spermatocytes we observed down regulation of *Piwil1* and *Piwil2*, while *Piwil4* expression was absent (it is expressed primarily in primordial germ cells; [67]). Although mutations for any of the three murine PIWI proteins confer male-specific sterility [35,68,69], the exact function of PIWIL1 and PIWIL2 proteins in adult testis is not known. They are a subset of the Argonaute proteins and associate specifically with small RNAs called PIWI-interacting RNAs (piRNAs) [70]. In the absence of PIWIL1 and PIWIL2 proteins, spermatocytes and spermatids are devoid of all piRNAs and spermatogenesis is terminally arrested during prophase I of meiosis [71].

PIWI proteins and piRNAs are enriched in the male germ-cell specific structures: the dense (sex) body and the chromatoid body. The dense body is associated with synapsis and the formation of the XY body during meiosis, however both processes are not affected in the absence of PIWI proteins [71]. The X and Y chromosomes in the dense body are transcriptionally silenced during meiosis at the pachytene stage [72], yet the sex chromosomal multicopy genes can escape the postmeiotic repression [73,74]. Because PIWIL2 probably participates in chromatin relaxation during DNA repair [75], one could speculate that it might also relax chromatin in sex chromosomes to enable transcription of such genes. It has been shown that expression of multicopy genes located in the dense body is regulated by HSF1 and HSF2 [55,76]. Because either activation of HSF1 or simultaneous lack of both HSF1 and HSF2 are connected with the diminished fertility of males [16,17,77], one could speculate that disturbances in the transcription of the sex chromosomal multicopy genes could be at least one reason for HSF1-related fertility problems in males. The chromatoid body contains abundant RNA binding proteins and has been implicated in the storage, metabolism, and cytoplasm-to-nuclear transport of mRNA, small RNA, and related proteins [78]. Multiple members of the *tudor* domain containing proteins are also components of the chromatoid body and can associate with PIWI proteins. TDRD1 specifically interacts with PIWIL2 [34,36,79]. In addition, PIWIL2 positively regulates *Tdrd1* expression at the mRNA level [36]. It has been proposed that TDRD1 can act as a scaffold protein for complex assembly in the piRNA pathway [80]. MOV10L1 (a germ cell–specific putative RNA helicase) also associates with the PIWIL2-piRNA complex in adult testis [38,39,65]. Genetic disruption of the MOV10L1 RNA helicase domain in mice is connected with a lack of piRNAs and male sterility. Thus, down-regulation of *Piwil2*, *Tdrd1* and *Mov10l1* following heat shock is apparently connected with deregulated piRNA biogenesis.

Our study revealed that transcription of many genes important for spermatogenesis is negatively regulated following hyperthermia independently of HSF1 binding. It has been shown that lack of CDK2 [81], MLH3 [82], SYCP3 [83], MYBL1 [84], GJA1 [85], CLDN11 [86], BSG [87], POU5F2 [88], NRAS [89], CTSL [90], and possibly many others is connected with male sterility. We propose that repression of these genes (and other HSF1-independent genes) in cells subjected to hyperthermia results from activity of SPEN, which is specifically induced in heat shocked spermatocytes. SPEN is a large multidomain protein with ability to bind to DNA and RNA, and is a putative general transcriptional repressor . Importantly, SPEN can interact directly with SMRT (Silencing Mediator for Retinoid and Thyroid receptors; NCOR2) and members of the NuRD (nucleosome remodeling and deacetylating) complex including HDAC1 and HDAC2, MTA2, MBD3 and RbAp48 [91]. It was suggested that SPEN serves as a nuclear matrix platform that organizes and integrates transcriptional responses: recruits proteins involved in histone deacetylation, or acts by sequestration of transcriptional activators. Here we show that heat shock-induced up-regulation of *Spen* expression correlates with HSF1 binding to its promoter, which indicates its direct HSF1 dependence.

## Conclusions

Although in somatic and spermatogenic cells after activation HSF1 binds to many promoters in a similar way, its impact on genes’ expression is completely different. This results in a completely different heat shock response in these two types of cells. In hepatocytes, which are able to survive following heat shock, HSF1 activation is connected rather with up-regulated gene expression, whereas in spermatogenic cells, which induce apoptosis, HSF1 activation is connected with repression of the transcription. HSF1-dependent induction of apoptosis in spermatocytes seems to be caused by the simultaneous repression of many genes essential for spermatogenesis. Some of these genes could be down-regulated due to direct HSF1 binding, while repression of other genes is putatively an indirect effect of HSF1, executed by transcription repressors regulated by this factor. Hence, a broad range of other transcription factors, some of them induced by HSF1, could also contribute to cell-type specific mechanisms regulating transcriptional response to hyperthermia. The obtained results suggest that deregulated RNA (especially piRNA) metabolism could be the most important factor triggering spermatogenic cell death following heat shock.

## Methods

### Experimental animals and ethical statement

Adult (10 to 16-week-old), traditional inbred FVB/N male mice (mean weight: 27.6 g ± 2.1 g) were used for analyses (source: Animal Facility in the Cancer Center and Institute of Oncology, adherent to a high standard of veterinary care). The animals (typically 2–8 animals per cage; minimum floor space per animal – 100 cm^2^) were maintained under controlled environmental conditions with a 12 h light:12 h darkness cycle and were provided with food and tap water. The animal experiments were carried out according to Polish legislation, and were approved by the Local Committee of Ethics and Animal Experimentation at the Medical University of Silesia in Katowice, Poland (Decision No 82/2009) and by the institutional animal care policy of the Cancer Center and Institute of Oncology (Gliwice, Poland).

### Isolation of spermatocytes

The mice were killed by CO_2_ asphyxiation. The testes (twenty males for one isolation) were decapsulated and cell suspension was obtained by treatment with collagenase type IA (Sigma), trypsin and DNase I as described previously [92,93]. Population of cells enriched in spermatocytes (up to 80%) was obtained by unit gravity sedimentation in a Sta-Put chamber (SP-120, ProScience Inc, Ontario, Canada) containing a 1-3% (w/v) linear BSA gradient in PBS [94]. The isolated fraction of spermatocytes was contaminated mainly with round spermatids due to their tendency to form multinucleated cells. Over 95% of the cells were viable as assessed by the exclusion of trypan blue. The yield of spermatocytes prepared in one isolation was usually ~5×10^7^. Finally cells were suspended in CO_2_ saturated RPMI medium (1 × 10^6^ cells per 1 ml) supplemented with 10% (v/v) fetal bovine serum, 0.004% (v/v) gentamycin (KRKA) and 6mM sodium lactate (Sigma). After each isolation cells were equally divided for control group and heat shocked at 38°C or 43°C groups. Totally, 12 isolations were performed.

### Isolation of hepatocytes

Mouse hepatocytes were obtained from 10 to 16-week-old FVB/N males by the collagenase perfusion method [95]. Briefly, the liver was cannulated via the portal vein and perfused with 20 ml of warm (37°C) Krebs-Ringer buffer (5 mM HEPES, pH 7.4; 4,8 mM KCl; 1.2 mM MgSO4; 1.2 mM KH_2_PO_4_; 120 mM NaCl; 24 mM NaHCO_3_; 20 mM D-glucose) containing 0.5 mM EGTA at a rate of 5 ml/min with the perfusate exiting through the thoracic anterior vena cava. The liver was then perfused with a 20 ml of Krebs-Ringer buffer without EGTA, and then with a ~40 ml buffer containing 1.37 mM calcium chloride and 60–100 mg collagenase type IA (Sigma) for up to 10 min. After dispersion and washing in cold PBS (137 mM NaCl; 2.7 mM KCl; 8.1 mM Na_2_HPO_4_; 1.5 mM KH_2_PO_4_, pH 7.2), isolated hepatocytes were filtered through a 100-μm cell strainer. Cells were assessed for viability by trypan blue exclusion. A yield of 0.8 to 1×10^8^ was routinely obtained with over 93% viability. Isolated hepatocytes were resuspended in DMEM medium supplemented with 10% (v/v) fetal bovine serum, 0.004% (v/v) gentamycin (KRKA) and 1 μM insulin. Totally, 12 isolations were performed. For ChIP experiments, after each isolation cells were equally divided for control and heat shocked at 43°C group.

### Heat shock treatment

For global gene expression analyses spermatocytes from three independent isolations were heat shocked for one hour at 38°C or 43°C in a CO_2_ incubator and allowed to recover at the physiological temperature (32°C). Due to difficulties with the isolation of high quality intact RNA from hepatocytes after heat shock done *in vitro*, whole-body hyperthermia was performed *in vivo*. Animals were anesthetized using avertin (15–17 μl of a 2.5% solution/g body weight) injected intraperitoneally. The lower half of the torso of each animal was submerged in a water bath at 43°C for 30 min, after which time the animals were dried and returned to their cages. Hepatocytes were collected by collagenase perfusion to obtain cells ready for harvesting after two hours from heat shock. Cells were washed in cold PBS and frozen as a pellet at -70°C until RNA isolation. For RT-PCR analyses, organs (testes and livers) were collected either from control, untreated, or *in vivo* heat-shocked animals, and frozen on dry ice. For ChIP experiments, an equal volume (10 ml) of CO_2_ saturated, pre-heated media (to 55°C and 60°C for hepatocytes and spermatocytes, respectively) were added to the cell suspension, which immediately raised the temperature of the media to 42-43°C. The tubes were sealed with parafilm and submerged in a water bath at 43°C for an additional 5 to 40 min. Spermatocytes were heat shocked also at 38°C in a similar way except that the medium was pre-heated to 53°C. Immediately after heat shock cells were fixed by adding formaldehyde according to ChIP protocols. Simultaneously the medium was quickly cooled to room temperature.

### RNA isolation, cDNA synthesis and RT-PCR

For global gene expression analyses, the total RNA was purified in triplicate from isolated spermatocytes and hepatocytes using RNeasy® Mini Kit spin columns (Qiagen, Valencia, CA, USA). Validation of the RNA quality was performed using the RNA 6000 Nano Assay on an Agilent 2100 Bioanalyzer (Agilent Technologies, Palo Alto, CA, USA). For RT-PCR, the total RNA was isolated from independent samples (isolated spermatocytes, testes, livers) at least twice using the GeneMATRIX Universal RNA Purification Kit (Eurx, Gdańsk, Poland), and then 25–50 μg of RNA was incubated with 2.5 U of DNase I (Worthington Biochemical Corporation, NY, USA) for 15–30 min at 37°C in a buffer containing 40 mM Tris pH 7.9; 10 mM NaCl; 6 mM MgCl_2_; 0.1 mM CaCl_2_ to remove genomic DNA contamination. Reaction was stopped by heating for 5 min at 75°C and RNA was precipitated, washed, dissolved in RNase free water and tested for DNA contamination by PCR (35 cycles) with *Gapdh* primers. For cDNA synthesis 1 μg of RNA was primed with random pentadecamer primers (200 ng) [96] and 18-mer oligo-dT primers (250 ng) and the reaction was carried out at 37°C for 50 min in the presence of 5μM dNTPs and 200 U of reverse transcriptase (Gibco, Invitrogen) in a total volume of 20 μl. Reaction was terminated by heating at 70°C for 15 min, samples were diluted using water to 400 μl, aliquoted and stored at -20°C until use. For RT-PCR 1–3 μl of cDNA template was used and 14–35 cycles were applied depending on the primers set. For each primers set an optimal annealing temperature was established. Quantitative RT-PCR was performed using a Bio-Rad CFX 96™ Real-Time PCR Detection System. A total of 5 pmoles of forward and reverse primer, cDNA template, and Real-Time 2× PCR Master Mix SYBR A (A&A Biotechnology, Gdynia, Poland) were used. Reactions were incubated at 95°C for 3 min, followed by 40 cycles of 95°C for 25 sec, 61°C for 30 sec and 72°C for 30 sec. Analyzed genes were normalized against *Gapdh*. Primers used in analyses are listed in Additional file 17: Table S8 [24].

### Gene expression profiling by DNA microarrays

The global gene expression analysis was performed using the Affymetrix GeneChip® Mouse Genome 430 2.0 Array and GeneChip® Expression 3′Amplification reagents (Affymetrix, Santa Clara, CA) according to manufacturer’s protocols if not stated otherwise. The total RNA was extracted (from three independent samples from each cell type and each experimental point) as described above. cDNA was synthesized from 8 μg of total RNA using the One-Cycle cDNA Synthesis Kit with SuperScriptII polymerase then reverse-transcribed with a T7 RNA polymerase incorporating biotin-labeled nucleotides using the GeneChip IVT Labeling Kit. Biotin-labeled cRNA probe samples were purified, fragmented and hybridized (hybridization cocktail was prepared without DMSO) at 45°C overnight to the array. The arrays were washed, stained and scanned using a Fluidics Station and microarray scanner, which are components of the Affymetrix GeneChip Instrument System. Analyses were done in triplicate. Data was analyzed with R/Bioconductor and Matlab softwares, RMA (Robust Multi-array Analysis) algorithm with Dai (ver. 15.1) annotation were used for data normalization [97,98]. The level of genes expression was shown in arbitrary units in the logarithmic scale (log2). Noise threshold was estimated by modeling the signal distribution as Gaussian mixture (GM) and defining the first component as a model of noise. The remaining GM components were treated as the models of low and high signals (Additional file 1: Table S1 [24]). Changes in the level of genes expression in heat-shocked samples versus control, untreated, were presented as a signal log ratio (SLR, the average value of HS/average value of C). The GM model applied to SLR distribution allowed for identification of significantly up and down regulated genes (Table 1). Gene clustering based on temperature-related changes was performed with the use of multidimensional GM modeling [99] and the obtained group of genes were tested for overrepresentation of GO terms with the use of conditional hypergeometric test. Characteristics of individual genes biological functions were made on the basis of information available on the websites: [100,101].

### Chromatin Immunoprecipitation (ChIP) Assays and ChIP on chip

For analyses of HSF1 binding kinetics, the ChIP assay was carried out using a ChIP kit (Upstate Biotechnology, Lake Placid, NY) following the manufacturer’s protocol. To obtain recommended shared DNA size fragments (between 200–1,000 bp) chromatin from heat shocked samples had to be sonicated using two times more pulses than chromatin from control samples. For 1 × 10^6^ cells, 0.3 to 3 μg of rabbit anti-HSF1 polyclonal antibody (SPA-901, Stressgen, Victoria, Canada) was tested with similar qualitative results. Immunoprecipitated DNA was analyzed by PCR (ChIP-PCR). Primers characteristics used in analyses are presented in Additional file 18: Table S9 [24]. ChIP on chip analyses were done according to the Affymetrix protocol [102], with one modification: in the second round of immunoprecipitated DNA amplification, 2 mM magnesium chloride was applied instead of the recommended 0.75 mM. For one immunoprecipitation (IP), 5 × 10^7^ cells and 15 μg of anti-HSF1 antibody (SPA-901, Stressgen) were used (sample marked as AB1). A negative control was performed using the same number of cells with no antibody (mock IP, sample marked as AB0). Immunoprecipitated DNA was analyzed on the GeneChip® Mouse Promoter 1.0R Array. ChIP on chip analyses were done in triplicate. Data was quantile normalized with the use of dCHIP software and Mm_PromPR_v02-1_NCBIv36.NR.bpmap file. 5th percentile was used for background subtraction (PM-only). The further analysis was done in Matlab environment, the subsets of probes related to the regions defined by gene Accession Number were analyzed individually. The statistical tests for signal enhancement within the region and nonuniformity of the signal AB1-AB0 difference distribution were performed. Ontology of HSF1 bound genes was analyzed by NucleoAnnot application created within the confines of the GENEPI LowRT project (available on [103]).

### Availability of supporting data

Expression microarray and ChIP on chip data discussed in this article have been deposited in NCBI’s Gene Expression Omnibus [104] and are accessible through GEO Series accession number GSE40248 and GSE40390. The data sets supporting the results of this article are available in the LabArchives repository [24].

## Abbreviations

AB1/AB0: ChIP samples precipitated with/without antibody; BSA: Bovine serum albumin; C: Control; ChIP on chip: Chromatin immunoprecipitation assay combined with promoter microarrays; ChIP-PCR: Chromatin immunoprecipitation followed by PCR; ChIP-seq: ChIP with massively parallel DNA sequencing; FDR: False discovery rate; GO: Gene ontology; GM: Gaussian mixture; HS: Heat-shock; HSPs: Heat shock proteins; HSF: Heat shock factor; RT-PCR: Reverse transcription polymerase chain reaction; RMA: Robust multi-array analysis; SLR: Signal log ratio.

## Competing interests

The authors declare that they have no competing interests.

## Authors’ contributions

MK-L carried out most the molecular biology experiments. JP performed bioinformatics analyses of expression and promoter microarrays, interpretation including annotation, GO analysis. JK participated in verification of microarrays data by RT-PCR and qPCR. MO hybridized samples on microarrays. NV participated in the ChIP experiments. AT participated in verification of microarrays data. WW conceived, designed, supervised, carried out analysis and wrote the manuscript. All authors read and approved the final manuscript.

## Supplementary Material

Additional file 1: Table S1 Noise threshold in expression microarrays. Signals are in arbitrary units in log2 scale. Available at: https://mynotebook.labarchives.com/share/HSF1%2520in%2520SC%2520and%2520HEP/MjIuMXwxMjY2MS8xNy02L1RyZWVOb2RlLzI3Nzc1NTE3NjV8NTYuMQ. Click here for file

Additional file 2 **Data from the global gene expression analyses.** Excel document contains all data from expression microarrays studies after normalization according to Dai et al. [97]. Legends for abbreviations used in headings are given in comments fields. Available at: https://mynotebook.labarchives.com/share/HSF1%2520in%2520SC%2520and%2520HEP/MjAuOHwxMjY2MS8xNi00L1RyZWVOb2RlLzM3NDU1NjIyM3w1Mi44. Click here for file

Additional file 3: Table S2 Top ten genes identified in spermatocytes (SC) and hepatocytes (HEP) as the most induced (red bold) by heat shock (at 38°C or 43°C) versus control (C). The level of expression is given in arbitrary units in logarithmic scale (log2). Changes in gene expression are shown as SLR. Available at: https://mynotebook.labarchives.com/share/HSF1%2520in%2520SC%2520and%2520HEP/MjMuNHwxMjY2MS8xOC04L1RyZWVOb2RlLzE5MTA1NDEzMDJ8NTkuNA. Click here for file

Additional file 4 **Cluster analysis in spermatocytes subjected to heat shock at 38°C and biological processes linked to genes grouped in clusters.** Compressed file contains: Excel document with “cluster analysis_summary” sheet (clusters with genes positively regulated following heat shock are marked in red, with genes negatively regulated – in green), and “cluster analysis_all” sheet with all genes shown separately. Legends for abbreviations used in headings are given in comments fields; a folder with results of *Gene to GO BP* Conditional test for over-representation analyzed in each cluster (html files). Available at: https://mynotebook.labarchives.com/share/HSF1%2520in%2520SC%2520and%2520HEP/MjQuN3wxMjY2MS8xOS0xMC9UcmVlTm9kZS8zMzM5NjMwMjc1fDYyLjc. Click here for file

Additional file 5 **Cluster analysis in spermatocytes subjected to heat shock at 43°C and biological processes linked to the genes grouped in clusters.** File contents – as in file 4. Available at: https://mynotebook.labarchives.com/share/HSF1%2520in%2520SC%2520and%2520HEP/MjYuMHwxMjY2MS8yMC0xMi9UcmVlTm9kZS8xMjU4MDk0NDYwfDY2LjA. Click here for file

Additional file 6 **Cluster analysis in hepatocytes subjected to the heat shock at 43°C and biological processes linked to the genes grouped in clusters.** File contents – as in file 4. Available at: https://mynotebook.labarchives.com/share/HSF1%2520in%2520SC%2520and%2520HEP/MjcuM3wxMjY2MS8yMS0xNC9UcmVlTm9kZS8zOTM2OTU2MjV8NjkuMw. Click here for file

Additional file 7: Table S3 Top ten genes identified in spermatocytes (SC) and hepatocytes (HEP) as the most repressed (green bold) by heat shock (at 38°C or 43°C) versus control (C). The level of expression is given in arbitrary units in logarithmic scale (log2). Changes in gene expression are shown as SLR. Available at: https://mynotebook.labarchives.com/share/HSF1%2520in%2520SC%2520and%2520HEP/MjguNnwxMjY2MS8yMi0yNi9UcmVlTm9kZS8xNzcwMjQwNTY0fDcyLjY. Click here for file

Additional file 8: Table S4 Genes whose expression was changed in the same direction following hyperthermia in spermatocytes (SC) and in hepatocytes (HEP) (at 38°C or 43°C, respectively). The level of expression is given in arbitrary units in logarithmic scale (log2). Changes in gene expression are shown as SLR (up-regulation: red bold, down-regulation: green bold). Available at: https://mynotebook.labarchives.com/share/HSF1%2520in%2520SC%2520and%2520HEP/MjkuOXwxMjY2MS8yMy0yNy9UcmVlTm9kZS80NDcyMjM4MDZ8NzUuOQ. Click here for file

Additional file 9: Figure S1 Detection of transcripts of selected genes in isolated spermatocytes, testes and liver up to 24h after hyperthermia, by RT-PCR. (**A**) Expression of selected genes connected with cell death shown in the main text in Table 3, (**B**) transcription factors shown in Table 4, (**C**) genes involved in inflammatory and immune responses shown in Table 5, (**D**) two uncharacterized genes induced in spermatocytes at 38°C, and (**E**) reference genes. N, PCR negative control without template. Available at: https://mynotebook.labarchives.com/share/HSF1%2520in%2520SC%2520and%2520HEP/MzEuMnwxMjY2MS8yNC0yOC9UcmVlTm9kZS8yMDQ4NTQ5MjI1fDc5LjI. Click here for file

Additional file 10 **Data from ChIP on chip analyses combined with the results of gene expression analyses.** Legends for abbreviations used in headings are given in the comments fields. Available at: https://mynotebook.labarchives.com/share/HSF1%2520in%2520SC%2520and%2520HEP/MzIuNXwxMjY2MS8yNS0yOS9UcmVlTm9kZS8xNjM2MjMxMjA0fDgyLjU. Click here for file

Additional file 11: Table S5 Top genes with the uppermost HSF1 binding in spermatocytes and hepatocytes at control temperature and following heat shock Binding of HSF1 is expressed as a mean AB1-AB0 value in arbitrary units. Genes marked in blue in group A are present also in groups B and C, marked in blue in group B are present also in group C. Available at: https://mynotebook.labarchives.com/share/HSF1%2520in%2520SC%2520and%2520HEP/MzMuOHwxMjY2MS8yNi0zMC9UcmVlTm9kZS8zMTQ1MjU5MzIxfDg1Ljg. Click here for file

Additional file 12 **Gene ontology analysis of genes bound by HSF1 in spermatocytes and hepatocytes following heat shock.** Excel documents contain: -sheet 1: gene to GO terms association; -sheet 2: statistics of biological processes (BP) linked to HSF1-bound genes; -sheet 3: statistics of cellular components (CC) linked to HSF1-bound genes; -sheet 4: statistics of molecular functions (MF) linked to HSF1-bound genes. Terms with the biggest representations were marked in red. Available at: https://mynotebook.labarchives.com/share/HSF1%2520in%2520SC%2520and%2520HEP/NDkuNHwxMjY2MS8zOC01My9UcmVlTm9kZS8xOTEyNDE5MDI0fDEyNS40. Click here for file

Additional file 13 **Correlation of HSF1 binding with changes of gene expression following heat shock.** Excel document contains lists of genes bound by HSF1 and: -sheet 1: up-regulated in spermatocytes at 38°C;-sheet 2: down-regulated in spermatocytes at 38°C;-sheet 3: up-regulated in spermatocytes at 43°C;-sheet 4: down-regulated in spermatocytes at 43°C;-sheet 5: up-regulated in hepatocytes at 43°C;-sheet 6: down-regulated in hepatocytes at 43°C. For HSF1 binding values AB1-AB0 ≥ 15 and FDR < 0.125 were taken into consideration (values 0.05 < FDR < 0.125 are shown in grey); in the case of changes in expression level – range of SLR presented in Table 1 in the main text. Legends for abbreviations used in headings are given in the comments fields. Available at: https://mynotebook.labarchives.com/share/HSF1%2520in%2520SC%2520and%2520HEP/MzYuNHwxMjY2MS8yOC0zMi9UcmVlTm9kZS8yODM3ODU2MzAxfDkyLjQ. Click here for file

Additional file 14: Table S6 Correlation between hyperthermia-induced changes in gene expression and HSF1 binding to promoters in control (C) or heat-shocked (HS) cells. All genes with expression above the noise threshold and SLR ≠ 0, and with binding of HSF1 where AB1 > AB0 were included into analyzes (N); correlations were estimated using the Spearman’s rank correlation coefficient (rho). Available at: https://mynotebook.labarchives.com/share/HSF1%2520in%2520SC%2520and%2520HEP/MzcuN3wxMjY2MS8yOS0zMy9UcmVlTm9kZS85Njk2ODI0NTV8OTUuNw. Click here for file

Additional file 15: Table S7 Characteristics of genes whose repression following heat shock in spermatocytes correlates with HSF1 binding. Genes necessary for spermatogenesis listed in the main text in Table 9 are marked here in red. Available at: https://mynotebook.labarchives.com/share/HSF1%2520in%2520SC%2520and%2520HEP/MzkuMHwxMjY2MS8zMC0zNC9UcmVlTm9kZS8zODY1NTg0NzMxfDk5LjA. Click here for file

Additional file 16: Figure S2 Changes of expression of selected genes in spermatocytes following heat shock at 38°C (one hour and two hours of recovery) assessed by quantitative RT-PCR. Values are shown in arbitrary units and are calculated against the level of expression at a physiological temperature which is 1.0. Expression was normalized against the level of *Gapdh*. *p-value > 0.05. Available at: https://mynotebook.labarchives.com/share/HSF1%2520in%2520SC%2520and%2520HEP/NDAuM3wxMjY2MS8zMS0zNS9UcmVlTm9kZS8yMjk1MDc0NDUzfDEwMi4z. Click here for file

Additional file 17: Table S8 Characteristics of primers used in RT-PCR analyses. Available at: https://mynotebook.labarchives.com/share/HSF1%2520in%2520SC%2520and%2520HEP/NDQuMnwxMjY2MS8zNC00Mi9UcmVlTm9kZS8yMDM3OTUxNzR8MTEyLjI. Click here for file

Additional file 18: Table S9 Characteristics of primers used in ChIP-PCR analyses. Available at: https://mynotebook.labarchives.com/share/HSF1%2520in%2520SC%2520and%2520HEP/NDIuOXwxMjY2MS8zMy0zOC9UcmVlTm9kZS80NTMxNzgxMTR8MTA4Ljk. Click here for file
