# Supplementary material for: Impact of heat shock transcription factor 1 on global gene expression profiles in cells which induce either cytoprotective or pro-apoptotic response following hyperthermia
Source: BMC Genomics. 2013 Jul 8;14:456. doi: 10.1186/1471-2164-14-456 (PMC3711851; doi:10.1186/1471-2164-14-456)
Supplement: Additional file 1: Table S1 — Noise threshold in expression microarrays. Signals are in arbitrary units in log2 scale. Available at: https://mynotebook.labarchives.com/share/HSF1%2520in%2520SC%2520and%2520HEP/MjIuMXwxMjY2MS8xNy02L1RyZWVOb2RlLzI3Nzc1NTE3NjV8NTYuMQ. [file 1471-2164-14-456-S1.docx]

**Table S1. Noise threshold in expression microarrays.** Signals are in arbitrary units in log2 scale

|  | Spermatocytes | | | Hepatocytes | |
| --- | --- | --- | --- | --- | --- |
|  | Control | HS_38 | HS_43 | Control | HS_43 |
| Noise | below 4.50 | below 4.36 | below 4. 53 | below 4.47 | below 4.49 |
| High | above 7.78 | above 7.92 | above 7.79 | above 8.80 | above 8.80 |
