# Supplementary material for: Impact of heat shock transcription factor 1 on global gene expression profiles in cells which induce either cytoprotective or pro-apoptotic response following hyperthermia
Source: BMC Genomics. 2013 Jul 8;14:456. doi: 10.1186/1471-2164-14-456 (PMC3711851; doi:10.1186/1471-2164-14-456)
Supplement: Additional file 3: Table S2 — Top ten genes identified in spermatocytes (SC) and hepatocytes (HEP) as the most induced (red bold) by heat shock (at 38°C or 43°C) versus control (C). The level of expression is given in arbitrary units in logarithmic scale (log2). Changes in gene expression are shown as SLR. Available at: https://mynotebook.labarchives.com/share/HSF1%2520in%2520SC%2520and%2520HEP/MjMuNHwxMjY2MS8xOC04L1RyZWVOb2RlLzE5MTA1NDEzMDJ8NTkuNA. [file 1471-2164-14-456-S3.docx]

**Table S2. Top ten genes identified in spermatocytes (SC) and hepatocytes (HEP) as the most induced (red bold) by heat shock (at 38^0^C or 43^0^C) versus control (C).** The level of expression is given in arbitrary units in logarithmic scale (log2). Changes in gene expression are shown as SLR

|  | **Entrez Gene ID** | **Mean expression** | | | **SLR** | | **Mean expression** | | **SLR HEP_43 vs C** | **Gene symbol (full name)** |
| --- | --- | --- | --- | --- | --- | --- | --- | --- | --- | --- |
|  |  | **SC_C** | **SC_38** | **SC_43** | **SC_38 vs C** | **SC_43 vs C** | **HEP_C** | **HEP_43** |  |  |
| 1. **Spermatocytes, heat shock 38^0^C** | | | | | | | | | | |
| 1. | 67407 | 6.22 | 8.44 | 6.14 | **2.21** | -0.08 | nl | nl | - | *Cylc1* (cylicin, basic protein of sperm head cytoskeleton 1) |
| 2. | 433215 | 6.63 | 8.78 | 6.72 | **2.15** | 0.09 | nl | nl | - | *BC048609* |
| 3. | 74862 | 5.36 | 7.43 | 5.28 | **2.07** | -0.08 | nl | nl | - | *4930447F04Rik* |
| 4. | 66780 | 6.82 | 8.83 | 6.93 | **2.00** | 0.11 | nl | nl | - | *4933436I01Rik* |
| 5. | 114672 | 6.68 | 8.67 | 7.04 | **1.99** | 0.36 | nl | nl | - | *1700007E05Rik* |
| 6. | 70936 | 4.78 | 6.69 | 4.75 | **1.91** | -0.03 | nl | nl | - | 4931400O07Rik |
| 7. | 12386 | 5.11 | 6.89 | 5.05 | **1.78** | -0.06 | nl | nl | - | *Ctnna2* (catenin (cadherin associated protein), alpha 2) |
| 8. | 69476 | 5.75 | 7.50 | 5.86 | **1.74** | 0.11 | nl | nl | - | *1700029M03Rik* |
| 9. | 100503001 | 5.78 | 7.49 | 5.80 | **1.72** | 0.02 | 4.57 | 4.61 | 0.04 | *LOC100503001* |
| 10. | 75811 | 8.20 | 9.91 | 8.11 | **1.71** | -0.09 | 4.52 | 4.73 | 0.21 | *Fam154a* (family with sequence similarity 154, member A) |
| 1. **Spermatocytes, heat shock 43^0^C** | | | | | | | | | | |
| 1. | 15511 | 4.96 | 5.03 | 7.39 | 0.07 | **2.43** | 9.55 | 13.22 | 3.67 | *Hspa1b* (heat shock protein 1B) |
| 2. | 193740 | 5.86 | 6.46 | 7.36 | 0.59 | **1.50** | 6.93 | 13.10 | 6.17 | *Hspa1a* (heat shock protein 1A) |
| 3. | 14608 | 5.93 | 6.28 | 6.53 | 0.35 | **0.60** | 5.78 | 5.91 | 0.13 | *Gpr83* (G protein-coupled receptor 83) |
| 4. | 239410 | 6.86 | 6.74 | 7.46 | -0.12 | **0.60** | 6.61 | 6.95 | 0.33 | *A930017M01Rik* |
| 5. | 66789 | 5.91 | 6.,79 | 6.46 | 0.89 | **0.56** | 8.04 | 7.51 | -0.53 | *Alg14* (asparagine-linked glycosylation 14 homolog (yeast) |
| 6. | 17079 | 4,94 | 5,16 | 5,48 | 0,22 | **0,54** | 4,61 | 4,97 | 0,36 | *Cd180* (CD180 antigen) |
| 7. | 58185 | 5.42 | 6.49 | 5.96 | 1.07 | **0.54** | 5.89 | 5.78 | -0.11 | *Rsad2* (radical S-adenosyl methionine domain containing 2) |
| 8. | 434197 | 4.51 | 4.79 | 5.05 | 0.29 | **0.54** | 6.34 | 6.33 | -0.01 | *Fam169b* (family with sequence similarity 169, member B) |
| 9. | 100502687 | 6.19 | 6.30 | 6.70 | 0.11 | **0.51** | 6.24 | 6.38 | 0.13 | *LOC100502687* |
| 10. | 100503686 | 4.38^nl^ | 4.40 | 4.88 | 0.02 | **0.51** | 8.07 | 8.41 | 0.34 | *LOC100503686* |
| 1. **Hepatocytes, heat shock 43^0^C** | | | | | | | | | | |
| 1. | 193740 | 5.86 | 6.46 | 7.36 | 0.59 | 1.50 | 6.93 | 13.10 | **6.17** | *Hspa1a* (heat shock protein 1A) |
| 2. | 100302594 | nl | nl | nl | - | - | 5.06 | 10.43 | **5.37** | *Snord14e* (small nucleolar RNA, C/D box 14E) |
| 3. | 81489 | 11.40 | 10.78 | 11.52 | -0.62 | 0.12 | 8.48 | 12.88 | **4.40** | *Dnajb1* (DnaJ (Hsp40) homolog, subfamily B, member 1) |
| 4. | 11910 | 6.38 | 5.87 | 6.19 | -0.51 | -0.19 | 6.44 | 10.80 | **4.36** | *Atf3* (activating transcription factor 3) |
| 5. | 16476 | 5.86 | 4.75 | 5.72 | -1.11 | -0.14 | 8.41 | 12.47 | **4.07** | *Jun* (Jun oncogene) |
| 6. | 68763 | 8.19 | 8.26 | 8.05 | 0.07 | -0.14 | 7.09 | 10.91 | **3.82** | 1110038B12Rik |
| 7. | 58233 | 8.23 | 8.79 | 8.27 | 0.56 | 0.04 | 6.18 | 9.98 | **3.80** | *Dnaja4* (DnaJ (Hsp40) homolog, subfamily A, member 4) |
| 8. | 15511 | 4.96 | 5.03 | 7.39 | 0.07 | 2.43 | 9.55**^#^** | 13.22 | **3.67** | *Hspa1b* (heat shock protein 1B) |
| 9. | 29810 | 8.73 | 8.47 | 8.62 | -0.25 | -0.11 | 8.93 | 12.59 | **3.67** | Bag3 (BCL2-associated athanogene 3) |
| 10. | 59012 | nl | nl | nl | - | - | 3.93^nl^**^#^** | 7.52**^#^** | **3.59** | *Moxd1* (monooxygenase, DBH-like 1) |

nl – noise level; **^#^**high standard deviation (in a range 1.8 – 3.9)
