# Supplementary material for: Impact of heat shock transcription factor 1 on global gene expression profiles in cells which induce either cytoprotective or pro-apoptotic response following hyperthermia
Source: BMC Genomics. 2013 Jul 8;14:456. doi: 10.1186/1471-2164-14-456 (PMC3711851; doi:10.1186/1471-2164-14-456)
Supplement: Additional file 7: Table S3 — Top ten genes identified in spermatocytes (SC) and hepatocytes (HEP) as the most repressed (green bold) by heat shock (at 38°C or 43°C) versus control (C). The level of expression is given in arbitrary units in logarithmic scale (log2). Changes in gene expression are shown as SLR. Available at: https://mynotebook.labarchives.com/share/HSF1%2520in%2520SC%2520and%2520HEP/MjguNnwxMjY2MS8yMi0yNi9UcmVlTm9kZS8xNzcwMjQwNTY0fDcyLjY. [file 1471-2164-14-456-S7.docx]

**Table S3. Top ten genes identified in spermatocytes (SC) and hepatocytes (HEP) as the most repressed (green bold) by heat shock (at 38^0^C or 43^0^C) versus control (C).**

The level of expression is given in arbitrary units in logarithmic scale (log2). Changes in gene expression are shown as SLR

|  | **Entrez Gene ID** | **Mean expression** | | | **SLR** | | **Mean expression** | | **SLR HEP_43 vs C** | **Gene symbol (full name)** |
| --- | --- | --- | --- | --- | --- | --- | --- | --- | --- | --- |
|  |  | **SC_C** | **SC_38** | **SC_43** | **SC_38 vs C** | **SC_43 vs C** | **HEP_C** | **HEP_43** |  |  |
| 1. **Spermatocytes, heat shock 38^0^C** | | | | | | | | | | |
| 1. | 18643 | 10.41 | 7.57 | 10.16 | **-2.84** | -0.25 | 10.89 | 10.80 | -0.10 | *Pfn1* (profilin 1) |
| 2. | 432879 | 7.92 | 5.26 | 7.66 | **-2.66** | -0.26 | nl | nl | - | *Gm5465* |
| 3. | 71824 | 9.91 | 7.32 | 9.14 | **-2.59** | -0.76 | nl | nl | - | *1700006A11Rik* |
| 4. | 60406 | 9.25 | 6.78 | 9.16 | **-2.47** | -0.09 | 6.10 | 6.20 | 0.10 | *Sap30* (sin3 associated polypeptide) |
| 5. | 75939 | 10.12 | 7.74 | 9.52 | **-2.38** | -0.60 | 4.60 | 4.78 | 0.17 | *4930579G24Rik* |
| 6. | 100504457 | 10.01 | 7.67 | 9.59 | **-2.33** | -0.42 | 3.94^nl^ | 4.71 | 0.77 | *LOC100504457* |
| 7. | 194908 | 10.08 | 7.77 | 9.90 | **-2.30** | -0.18 | 4.70 | 4.73 | 0.03 | *Pld6* (phospholipase D family, member 6) |
| 8. | 74197 | 8.42 | 6.14 | 8.24 | **-2.28** | -0.18 | 6.83 | 7.04 | 0.21 | *Gtf2e1* (general transcription factor II E, polypeptide 1 (alpha subunit)) |
| 9. | 112418 | 10.65 | 8.39 | 10.26 | **-2.27** | -0.40 | 6.12 | 6.37 | 0.25 | *1700102P08Rik* |
| 10. | 26426 | 10.86 | 8.63 | 10.58 | **-2.23** | -0.28 | 8.41 | 8.58 | 0.18 | *Nubp2* (nucleotide binding protein 2) |
| 1. **Spermatocytes, heat shock 43^0^C** | | | | | | | | | | |
| 1. | 18417 | 5.36 | 3.96^nl^ | 4.01 | -1.40 | **-1.35** | nl | nl | - | *Cldn11* (claudin 11) |
| 2. | 13039 | 9.00 | 7.94 | 7.88 | -1.07 | **-1.13** | 11.26 | 11.04 | -0.22 | *Ctsl* (cathepsin L) |
| 3. | 11532 | 6.03 | 5.14 | 4.94 | -0.89 | **-1.09** | 12.30 | 12.18 | -0.11 | *Adh5* (alcohol dehydrogenase 5 (class III), chi polypeptide) |
| 4. | 246700 | 8.09 | 7.50 | 7.02 | -0.59 | **-1.07** | 4.68 | 4.75 | 0.07 | *Defb19* (defensin beta 19) |
| 5. | 50796 | 7.09 | 6.65 | 6.03 | -0.44 | **-1.06** | 4.50 | 4.33^nl^ | -0.17 | *Dmrt1* (doublesex and mab-3 related transcription factor 1) |
| 6. | 245688 | 7.22 | 6.51 | 6.22 | -0.71 | **-1.00** | 10.19 | 10.11 | -0.08 | *Rbbp7* (retinoblastoma binding protein 7) |
| 7. | 19716 | 5.14 | 4.47 | 4.16 | -0.68 | **-0.98** | nl | nl | - | *Bex1* (brain expressed gene 1) |
| 8. | 100038371 | 6.56 | 5.06 | 5.61 | -1.49 | **-0.95** | nl | nl | - | *Zfp389* (zinc finger protein 389) |
| 9. | 209416 | 5.78 | 5.83 | 4.84 | 0.05 | **-0.94** | 8.20 | 8.26 | 0.06 | *Gpkow* (G patch domain and KOW motifs) |
| 10. | 239435 | 8.25 | 7.32 | 7.31 | -0.93 | **-0.94** | 5.97 | 5.80 | -0.16 | *Aard* (alanine and arginine rich domain containing protein) |
| 1. **Hepatocytes, heat shock 43^0^C** | | | | | | | | | | |
| 1. | 13117 | nl | nl | nl | - | - | 8.25**^#^** | 4.63 | **-3.62** | *Cyp4a10* (cytochrome P450, family 4, subfamily a, polypeptide 10) |
| 2. | 13706 | 5.63 | 5.68 | 5.42 | 0.05 | -0.21 | 8.56**^#^** | 5.46 | **-3.10** | *Cela2a* (chymotrypsin-like elastase family, member 2A) |
| 3. | 109791 | nl | nl | nl | - | - | 6.64**^#^** | 3.98^nl^ | **-2.66** | *Clps* (colipase, pancreatic) |
| 4. | 69060 | 4.94 | 4.94 | 4.93 | 0.00 | -0.01 | 7.43**^#^** | 4.96 | **-2.47** | *Pnlip* (pancreatic lipase) |
| 5. | 109697 | 5.44 | 5.56 | 5.79 | 0.13 | 0.36 | 7.93**^#^** | 5.49 | **-2.44** | *Cpa1* (carboxypeptidase A1) |
| 6. | 112417 | nl | nl | nl | - | - | 7.47**^#^** | 5.41 | **-2.06** | *Ugt2b37* (UDP glucuronosyltransfera-se 2 family, polypeptide B37) |
| 7. | 76703 | 5.15 | 5.25 | 5.22 | 0.11 | 0.07 | 7.66**^#^** | 5.67 | **-2.00** | *Cpb1* (carboxypeptidase B1 (tissue)) |
| 8. | 66473 | 6.67 | 6.55 | 6.93 | -0.12 | 0.26 | 8.84**^#^** | 6.84 | **-2.00** | *Ctrb1* (chymotrypsinogen B1) |
| 9. | 19692 | 4.91 | 4.96 | 5.11 | 0.05 | 0.20 | 6.62**^#^** | 4.85 | **-1.77** | *Reg1* (regenerating islet-derived 1) |
| 10. | 109660 | 6.22 | 6.28 | 6.36 | 0.06 | 0.14 | 7.89**^#^** | 6.13 | **-1.77** | *Ctrl* (chymotrypsin-like) |

nl – noise level; **^#^**high standard deviation (in a range 1.4 – 3.6)
