# Supplementary material for: Impact of heat shock transcription factor 1 on global gene expression profiles in cells which induce either cytoprotective or pro-apoptotic response following hyperthermia
Source: BMC Genomics. 2013 Jul 8;14:456. doi: 10.1186/1471-2164-14-456 (PMC3711851; doi:10.1186/1471-2164-14-456)
Supplement: Additional file 8: Table S4 — Genes whose expression was changed in the same direction following hyperthermia in spermatocytes (SC) and in hepatocytes (HEP) (at 38°C or 43°C, respectively). The level of expression is given in arbitrary units in logarithmic scale (log2). Changes in gene expression are shown as SLR (up-regulation: red bold, down-regulation: green bold). Available at: https://mynotebook.labarchives.com/share/HSF1%2520in%2520SC%2520and%2520HEP/MjkuOXwxMjY2MS8yMy0yNy9UcmVlTm9kZS80NDcyMjM4MDZ8NzUuOQ. [file 1471-2164-14-456-S8.docx]

**Table S4. Genes whose expression was changed in the same direction following hyperthermia in spermatocytes (SC) and in hepatocytes (HEP) (at 38^0^C or 43^0^C, respectively).** The level of expression is given in arbitrary units in logarithmic scale (log2). Changes in gene expression are shown as SLR (up-regulation: red bold, down-regulation: green bold)

| **Lp.** | **Entrez Gene ID** | **Mean SC_C** | **Mean SC_38** | **SLR SC_38 vs C** | **Mean HEP_C** | **Mean HEP_43** | **SLR HEP_43 vs C** | **Gene Symbol (full name)** |
| --- | --- | --- | --- | --- | --- | --- | --- | --- |
| 1. | 22151 | 6.55 | 7.95 | **1.39** | 7.78 | 8.82 | **1.04** | *Tubb2a* (tubulin, beta 2A class IIA) |
| 2. | 218038 | 4.86 | 6.25 | **1.39** | 3.55 | 4.31 | **0.77** | *Amph* (amphiphysin) |
| 3. | 12192 | 6.09 | 7.03 | **0.94** | 10.05 | 11.26 | **1.21** | *Zfp36l1* (zinc finger protein 36, C3H type-like 1) |
| 4. | 26357 | 6.99 | 7.91 | **0.92** | 8.90 | 10.07 | **1.17** | *Abcg2* (ATP-binding cassette, sub-family G (WHITE), member 2) |
| 5. | 71724 | 7.89 | 8.80 | **0.91** | 11.37 | 12.33 | **0.95** | *Aox3* (aldehyde oxidase 3) |
| 6. | 20419 | 9.59 | 10.50 | **0.91** | 4.49 | 5.24 | **0.75** | *Shcbp1* (Shc SH2-domain binding protein 1) |
| 7. | 73363 | 7.45 | 8.31 | **0.86** | 5.04 | 5.90 | **0.87** | *1700056E22Rik* |
| 8. | 70893 | 9.15 | 9.98 | **0.83** | 3.89^nl^ | 4.84 | **0.95** | *Glb1l3* (galactosidase, beta 1 like 3) |
| 9. | 66961 | 7.75 | 6.26 | **-1.50** | 10.47 | 8.99 | **-1.48** | *Neat1* (nuclear paraspeckle assembly transcript 1 (non-protein coding)) |
| 10. | 13074 | 7.43 | 6.46 | **-0.97** | 7.10 | 5.71 | **-1.39** | *Cyp17a1* (cytochrome P450, family 17, subfamily a, polypeptide 1) |

nl – noise level
