# Supplementary figures and images for: Impact of heat shock transcription factor 1 on global gene expression profiles in cells which induce either cytoprotective or pro-apoptotic response following hyperthermia
Source: BMC Genomics. 2013 Jul 8;14:456. doi: 10.1186/1471-2164-14-456 (PMC3711851; doi:10.1186/1471-2164-14-456)

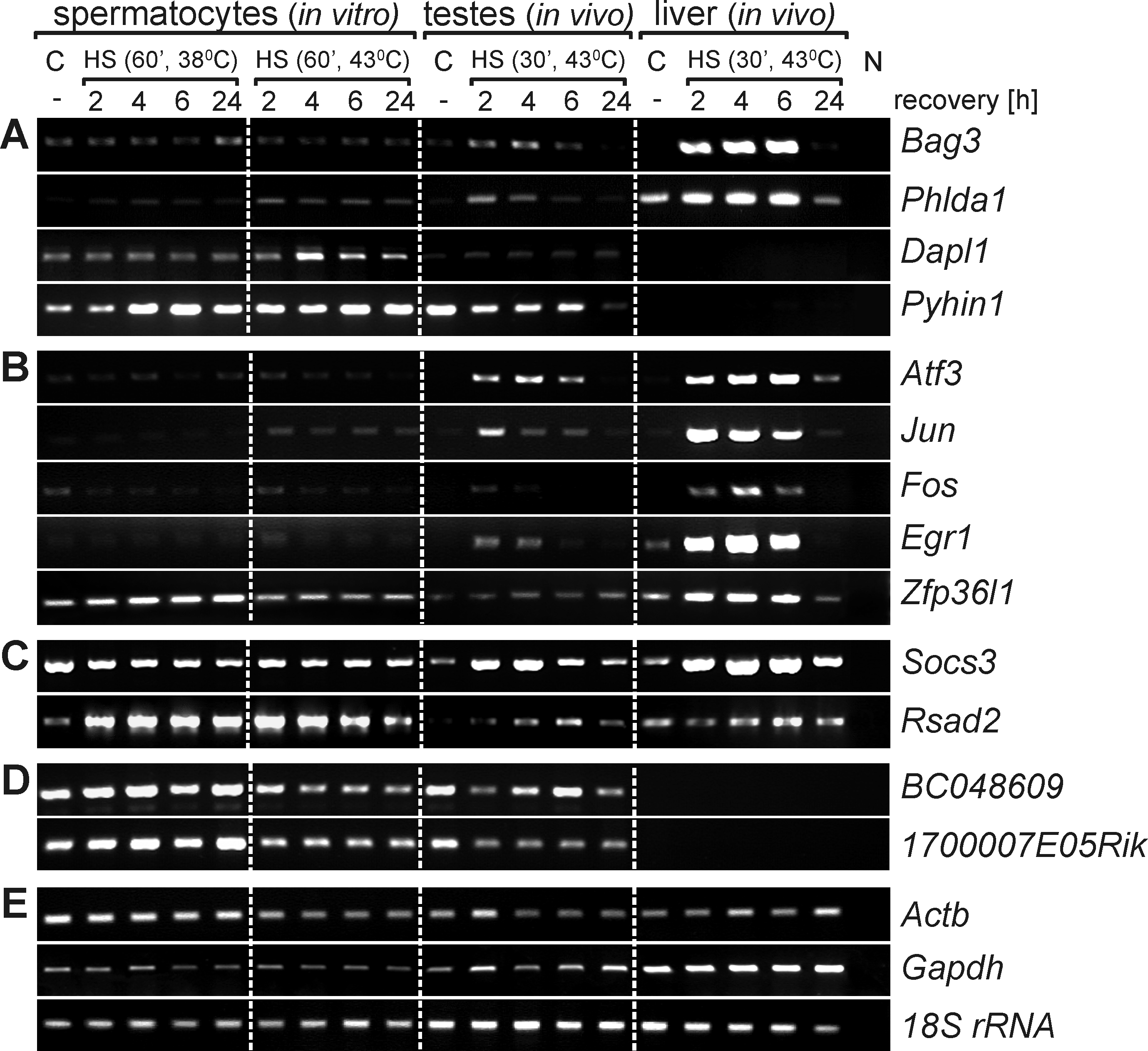

Supplement: Additional file 9: Figure S1 — Detection of transcripts of selected genes in isolated spermatocytes, testes and liver up to 24h after hyperthermia, by RT-PCR. (A) Expression of selected genes connected with cell death shown in the main text in Table 3, (B) transcription factors shown in Table 4, (C) genes involved in inflammatory and immune responses shown in Table 5, (D) two uncharacterized genes induced in spermatocytes at 38°C, and (E) reference genes. N, PCR negative control without template. Available at: https://mynotebook.labarchives.com/share/HSF1%2520in%2520SC%2520and%2520HEP/MzEuMnwxMjY2MS8yNC0yOC9UcmVlTm9kZS8yMDQ4NTQ5MjI1fDc5LjI. [file 1471-2164-14-456-S9.tiff]

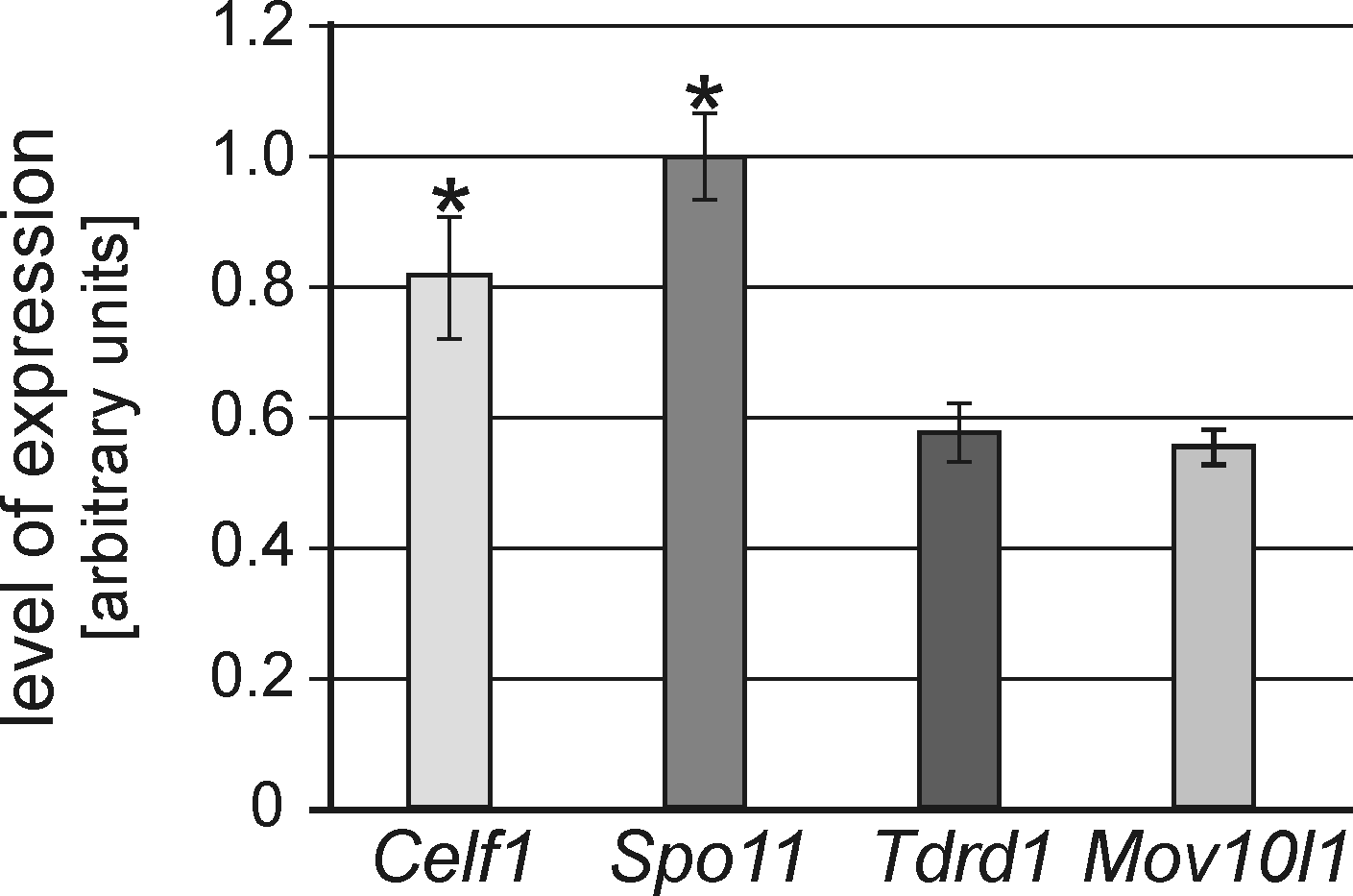

Supplement: Additional file 16: Figure S2 — Changes of expression of selected genes in spermatocytes following heat shock at 38°C (one hour and two hours of recovery) assessed by quantitative RT-PCR. Values are shown in arbitrary units and are calculated against the level of expression at a physiological temperature which is 1.0. Expression was normalized against the level of Gapdh. *p-value > 0.05. Available at: https://mynotebook.labarchives.com/share/HSF1%2520in%2520SC%2520and%2520HEP/NDAuM3wxMjY2MS8zMS0zNS9UcmVlTm9kZS8yMjk1MDc0NDUzfDEwMi4z. [file 1471-2164-14-456-S16.tiff]
