# Supplementary material for: Impact of heat shock transcription factor 1 on global gene expression profiles in cells which induce either cytoprotective or pro-apoptotic response following hyperthermia
Source: BMC Genomics. 2013 Jul 8;14:456. doi: 10.1186/1471-2164-14-456 (PMC3711851; doi:10.1186/1471-2164-14-456)
Supplement: Additional file 11: Table S5 — Top genes with the uppermost HSF1 binding in spermatocytes and hepatocytes at control temperature and following heat shock Binding of HSF1 is expressed as a mean AB1-AB0 value in arbitrary units. Genes marked in blue in group A are present also in groups B and C, marked in blue in group B are present also in group C. Available at: https://mynotebook.labarchives.com/share/HSF1%2520in%2520SC%2520and%2520HEP/MzMuOHwxMjY2MS8yNi0zMC9UcmVlTm9kZS8zMTQ1MjU5MzIxfDg1Ljg. [file 1471-2164-14-456-S11.docx]

**Table S5. Top genes with the uppermost HSF1 binding in spermatocytes and hepatocytes at control temperature and following heat shock.** Binding of HSF1 is expressed as mean AB1-AB0 value in arbitrary units. Genes marked in blue in group A are present also in groups B and C, marked in blue in group B are present also in group C

| **Accession No** | **Entrez Gene ID** | **Gene symbol** | **SC_C** | | **SC_38** | | **SC_43** | | **HEP_43** | |
| --- | --- | --- | --- | --- | --- | --- | --- | --- | --- | --- |
|  |  |  | **HSF1 binding** | **FDR** | **HSF1 binding** | **FDR** | **HSF1 binding** | **FDR** | **HSF1 binding** | **FDR** |
| 1. **Spermatocytes, control** | | | | | | | | | | |
| BC029834 | 108989 | ***Tpr*** | **30.22** | 0.0176 | **120.95** | 0.0000 | **133.26** | 0.0000 | 178.30 | 0.0000 |
| BC022652 | 100978 | ***Nfxl1*** | **28.78** | 0.0303 | **132.97** | 0.0000 | **122.85** | 0.0000 | 103.90 | 0.0000 |
| BC019693 | 217995 | *Heatr1* | **18.67** | 0.0488 | 72.47 | 0.0000 | 87.71 | 0.0000 | 51.43 | 0.0000 |
| BC059914 | 100302730 | ***5830417I10Rik*** | **17.64** | 0.0486 | **136.98** | 0.0000 | **165.65** | 0.0000 | 202.37 | 0.0000 |
| 1. **Spermatocytes, heat shock 38^0^C** | | | | | | | | | | |
| BC026480 | 24068 | *Sra1* | - | - | **271.10** | 0.0000 | 55.00 | 0.0005 | 500.54 | 0.0050 |
| AF388674 | 21871 | ***Atp6v0a2*** | - | - | **250.37** | 0.0001 | **202.62** | 0.0000 | 316.35 | 0.0002 |
| BC031519 | 226090 | ***Ermp1*** | 44.54 | 0.4165* | **219.11** | 0.0000 | **198.30** | 0.0000 | 222.03 | 0.0008 |
| BC030489 | 68743 | ***Anln*** | 17.09 | 0.6169* | **126.10** | 0.0000 | **124.82** | 0.0002 | 16.61 | 0.4392* |
| BC049797 | 235559 | *Topbp1* | - | - | **122.90** | 0.0029 | 96.50 | 0.0003 | 160.08 | 0.2782* |
| BC118968 | 319675 | *5830418K08Rik* | 19.09 | 0.1271* | **112.32** | 0.0000 | 118.96 | 0.0000 | 59.33 | 0.0000 |
| BC016616 | 76022 | ***Gon4l*** | 18.19 | 0.4463* | **111.17** | 0.0000 | **144.22** | 0.0000 | 190.33 | 0.0000 |
| 1. **Spermatocytes, heat shock 43^0^C** | | | | | | | | | | |
| BC027375 | 56085 | *Ubqln1* | - | - | 45.02 | 0.0001 | **154.48** | 0.0000 | - | - |
| BC017678 | 19731 | *Rgl1* | - | - | - | - | **145.86** | 0.0181 | - | - |
| BC003843 | 70356 | *St13* | - | - | 32.52 | 0.005 | **132.91** | 0.0000 | 99.84 | 0.0028 |
| 1. **Hepatocytes, heat shock 43^0^C** | | | | | | | | | | |
| AF093677 | 100191075 | *Gm10925* | - | - | - | - | - | - | **822.75** | 0.0000 |
| BC100413 | 100041223 | *LOC100041223^#^* | 24.15 | 0,8769* | - | - | - | - | **506.80** | 0.0000 |
| BC089465 | 434935 | *MGC107098^#^* | - | - | - | - | - | - | **506.62** | 0.0000 |
| BC026480 | 24068 | *Sra1* | - | - | 271.10 | 0.0000 | 55.00 | 0.0005 | **500.54** | 0.0050 |
| BC117086 | 70009 | *Ssty2^#^* | - | - | - | - | - | - | **496.10** | 0.0000 |
| BC100414 | 100040744 | *LOC100040744^#^* | - | - | - | - | - | - | **429.74** | 0.0000 |
| L04849 | 100039574 | *LOC100039574^#^* | - | - | - | - | - | - | **405.46** | 0.0002 |
| M38248 | 100039753 | *LOC100039753^#^* | - | - | - | - | - | - | **388.17** | 0.0000 |
| L04848 | 434960 | *LOC434960^#^* | - | - | - | - | - | - | **384.73** | 0.0004 |
| BC040090 | 100036568 | *Gm3373* | - | - | 96.15 | 0.0156 | - | - | **358.21** | 0.0006 |

*FDR>0.05; ^#^ binding on the Y chromosome
