# Supplementary material for: Impact of heat shock transcription factor 1 on global gene expression profiles in cells which induce either cytoprotective or pro-apoptotic response following hyperthermia
Source: BMC Genomics. 2013 Jul 8;14:456. doi: 10.1186/1471-2164-14-456 (PMC3711851; doi:10.1186/1471-2164-14-456)
Supplement: Additional file 14: Table S6 — Correlation between hyperthermia-induced changes in gene expression and HSF1 binding to promoters in control (C) or heat-shocked (HS) cells. All genes with expression above the noise threshold and SLR ≠ 0, and with binding of HSF1 where AB1 > AB0 were included into analyzes (N); correlations were estimated using the Spearman’s rank correlation coefficient (rho). Available at: https://mynotebook.labarchives.com/share/HSF1%2520in%2520SC%2520and%2520HEP/MzcuN3wxMjY2MS8yOS0zMy9UcmVlTm9kZS85Njk2ODI0NTV8OTUuNw. [file 1471-2164-14-456-S14.docx]

**Table S6. Correlation between hyperthermia-induced changes in gene expression and HSF1 binding to promoters in control (C) or heat-shocked (HS) cells**. All genes with expression above the noise threshold and SLR≠0, and with binding of HSF1 where AB1>AB0 were included into analyzes (N); correlations were estimated using the Spearman’s rank correlation coefficient (rho)

|  | N | Correlation with HSF1 binding at C | | Correlation with HSF1 binding at HS | | Correlation with the change in binding (HS vs C) | |
| --- | --- | --- | --- | --- | --- | --- | --- |
|  |  | rho | p-value | rho | p-value | rho | p-value |
| Up-regulated genes (SLR>0) | | | | | | | |
| Spermatocytes HS_38^0^C | 7032 | -0.003 | 7.71E-01 | -0.072 | 1.01E-09 | -0.058 | 1.16E-06 |
| Spermatocytes HS_43^0^C | 6906 | 0.008 | 4.71E-01 | -0.097 | 6.23E-16 | -0.102 | 2.33E-17 |
| Hepatocytes HS_43^0^C | 6798 | 0.008 | 4,78E-01 | 0.047 | 9.95E-05 | 0.036 | 3.11E-03 |
| Down-regulated genes (SLR<0) | | | | | | | |
| Spermatocytes HS_38^0^C | 6568 | -0.009 | 1.69E-02 | -0.138 | 2.89E-29 | -0.112 | 7.95E-20 |
| Spermatocytes HS_43^0^C | 6600 | 0.004 | 7.06E-01 | -0.066 | 8.32E-08 | -0.068 | 3.41E-08 |
| Hepatocytes HS_43^0^C | 6772 | -0.005 | 6.76E-01 | -0.032 | 7.66E-03 | -0.025 | 4.07E-02 |
