# Supplementary material for: Impact of heat shock transcription factor 1 on global gene expression profiles in cells which induce either cytoprotective or pro-apoptotic response following hyperthermia
Source: BMC Genomics. 2013 Jul 8;14:456. doi: 10.1186/1471-2164-14-456 (PMC3711851; doi:10.1186/1471-2164-14-456)
Supplement: Additional file 15: Table S7 — Characteristics of genes whose repression following heat shock in spermatocytes correlates with HSF1 binding. Genes necessary for spermatogenesis listed in the main text in Table 9 are marked here in red. Available at: https://mynotebook.labarchives.com/share/HSF1%2520in%2520SC%2520and%2520HEP/MzkuMHwxMjY2MS8zMC0zNC9UcmVlTm9kZS8zODY1NTg0NzMxfDk5LjA. [file 1471-2164-14-456-S15.docx]

**Table S7. Characteristics of genes whose repression following heat shock in spermatocytes correlates with HSF1 binding.**

Genes necessary for spermatogenesis listed in the full text in Table 9 are marked in red

| **Gene symbol (full name)** | **SLR** | | | **HSF1 binding** | | | **Function** |
| --- | --- | --- | --- | --- | --- | --- | --- |
|  | **SC_38 vs C** | **SC_43 vs C** | **HEP_ 43 vs C** | **SC_38 AB1-AB0** | **SC_43 AB1-AB0** | **HEP_43 AB1-AB0** |  |
| **Genes involved in DNA replication and cell division** | | | | | | | |
| *6330503K22Rik* (RIKEN cDNA 6330503K22 gene) | -2.16 | 0.03 | 0.56 | 66.0 | 82.0 | 27.3 | Necessary for centrosome duplication, required for correct spindle formation and has a role in regulating cytokinesis and genome stability. |
| *Zwilch* (Zwilch, kinetochore associated, homolog (Drosophila)) | -1.96 | -0.26 | -0.08 | 17.0 | 22.3 | 44.5 | Essential component of the mitotic checkpoint. |
| *Topbp1* (topoisomerase (DNA) II binding protein 1) | -1.57 | -0.54 | 0.11 | 122.9 | 96.5 | 160.1* | Required for DNA replication. Plays a role in the rescue of stalled replication forks and checkpoint control. |
| *Specc1l* (sperm antigen with calponin homology and coiled-coil domains 1-like) | -1.31 | -0.22 | -0.08 | 82.8 | 113.7 | 46.8 | Involved in cytokinesis and spindle organization. |
| *Spo11* (sporulation protein, meiosis-specific, SPO11 homolog (S. cerevisiae)) | -1.19 | 0.07 | -0.03 | 33.0 | 53.3 | - | Required for meiotic recombination (Baudat et al. 2000; Romanienko and Camerini-Otero 2000). |
| *Chordc1* (cysteine and histidine-rich domain (CHORD)-containing, zinc-binding protein 1) | -1.15 | 0.37 | 0.50 | 32.4 | 56.9 | 85.3* | Regulates centrosome duplication, Involved in stress response. Prevents tumorigenesis. |
| *Cdca8* (cell division cycle associated 8) | -1.06 | -0.18 | 0.06 | 79.6 | 42.3 | 97.0 | Required for chromatin-induced microtubule stabilization and spindle formation. |
| *Wdr92* (WD repeat domain 92) | -0.96 | -0.10 | -0.22 | 38.2 | 34.4 | 34.7* | Probably acts as a modulator of apoptosis. Proteins containing WD40 domains have a diverse range of functions, including signal transduction, cell cycle regulation, RNA splicing, and transcription. |
| *Cdk1* (cyclin-dependent kinase 1) | -0.98 | -0.15 | 0.40 | 21.8 | 21.2 | 13.3 | Regulatory roles in cell cycle control |
| *Numa1* (nuclear mitotic apparatus protein 1) | -0.97 | -0.11 | 0.09 | 25.6 | 35.2 | 40.6 | An essential tether linking bulk microtubules of the spindle to centrosome. |
| *Sfi1* (Sfi1 homolog, spindle assembly associated (yeast)) | -0.95 | -0.36 | -0.24 | 61,2 | - | 70.1* | Plays a role in the dynamic structure of centrosome-associated contractile fibers. |
| *Hells* (helicase, lymphoid specific) | -0.92 | -0.59 | 0.72 | 42.8 | 56.4 | 5.6* | Involved with cellular proliferation. |
| **Genes involved in regulation of the transcription** | | | | | | | |
| *Zc3h6* (zinc finger CCCH-type containing 6) | -1.42 | -0.19 | -0.20 | 34.2 | 41.8 | - | May be involved in regulation of the transcription (by similarity). |
| *Pou6f1* (POU class 6 homeobox 1) | -1.33 | -0.10 | -0.01 | 37.7 | - | 51.8* | Transcription factor. |
| *Trrap* (transformation/transcription domain-associated protein1) | -1.21 | -0.32 | -0.10 | 54.8 | 78.9 | 85.6 | Role in epigenetic transcription activation, required for the mitotic checkpoint and normal cell cycle progression. |
| *Sp3* (Sp3 transcription factor) | -1.29 | -0.08 | -0.36 | 26.9 | 19.9 | - | Transcriptional factor that can act as an activator or repressor. |
| *Phf7* (PHD finger protein 7) | -1.04 | -0.05 | 0.03 | 16.9 | 38.9 | 19.1 | Transcriptional regulation of spermatogenesis. |
| *Jmjd1c* (jumonji domain containing 1C) | -0.96 | -0.17 | 0.33 | 77.5 | 96.6 | 46.3 | Histone demethylase, playing a central role in histone code. |
| *Zfml* (zinc finger, matrin-like) | -0.90 | -0.29 | 0.28 | 64,2 | 58,9 | 38,3 | Associated with packaging,transferring, or processing transcripts. |
| *Med23* (mediator complex subunit 23) | -0.89 | -0.24 | -0.28 | 41.6 | 56.9 | 59.2 | Required for transcriptional activation subsequent to the assembly of the preinitiation complex. |
| **Genes involved in RNA processing** | | | | | | | |
| *Pdcd7*(programmed cell death 7) | -1.68 | -0.33 | 0.40 | 20.6 | 26.4 | 31.3 | Component of U12-type spliceosome. |
| *Edc4* (enhancer of mRNA decapping 4) | -1.54 | -0.13 | 0.05 | 21.7 | 19.8 | 43.1 | In the process of mRNA degradation, seems to play a role in mRNA decapping. |
| *Setx* (senataxin) | -1,40 | -0,06 | -0.02 | 69.3 | 35.6 | 35.2 | May be involved in RNA maturation and in DNA double-strand breaks damage response generated by oxidative stress. |
| *Rbm27*(RNA binding motif protein 27) | -1.24 | -0.10 | 0.03 | 42.3 | 54.7 | 31.6 | May be involved in mRNA and rRNA processing, RNA export, and RNA stability. |
| *Prpf4b* (PRP4 pre-mRNA processing factor 4 homolog B (yeast)) | -1.11 | -0.04 | 0.26 | 63.9 | 60.9 | 47.7 | Involved in pre-mRNA splicing. Phosphorylates SF2/ASF splicing factor. |
| *Prpf18* (PRP18 pre-mRNA processing factor 18 homolog (yeast)) | -1.04 | -0.10 | -0.14 | 26.7 | 14.9 | 22.4 | Participates in the second step of pre-mRNA splicing. |
| *Celf1* (CUGBP, Elav-like family member 1) | -1.07 | -0.01 | 0.19 | 35.1 | 48.3 | 24.5 | Post-transcriptional regulation: pre-mRNA alternative splicing, mRNA translation and stability. Required for completion of spermatogenesis (Kress et al. 2007). |
| *Srek1* (splicing regulatory glutamine/lysine-rich protein 1) | -0.93 | -0.14 | -0.03 | 41.9 | 51.3 | 22.0 | Regulation of alternative splicing. |
| *Txnl4b* (thioredoxin-like 4B) | -0.87 | -0.08 | -0.07 | 27.0 | 19.3 | 20.4* | Essential role in pre-mRNA splicing. Required in cell cycle progression for S/G(2) transition. |
| **Genes coding for proteins involved in intracellular transport** | | | | | | | |
| *Sec23a* (Sec23 homolog A (S. cerevisiae)) | -1.52 | -0.15 | -0.28 | 26.7 | 33.6 | - | Involved in transport from the endoplasmic reticulum to the Golgi apparatus. |
| *Vps16* (vacuolar protein sorting 16 homolog (S. cerevisiae)) | -1.20 | -0.32 | -0.05 | 26.7 | 37.6 | - | May play a role in vesicle-mediated protein trafficking to lysosomal compartments. |
| *Dync1li2* (dynein, cytoplasmic 1 light intermediate chain 2) | -1.12 | -0.13 | 0.01 | 17.4 | 30.8 | - | Acts as a motor for the intracellular retrograde motility of vesicles and organelles along microtubules. May play a role in binding dynein to membranous organelles or chromosomes. |
| *Slc7a5* (solute carrier family 7 (cationic amino acid transporter, y+ system), member 5) | -1.01 | -0.11 | -0.13 | 17.7 | 36.8 | - | Involved in transport of large neutral amino acids. |
| *Srp68* (signal recognition particle 68kDa) | -1.01 | -0.12 | 0.04 | 38.1 | 61.1 | 43.8 | Role in targeting secretory proteins to the rough  endoplasmic reticulum membrane. |
| *Kpnb1* (karyopherin (importin) beta 1) | -0.89 | -0.18 | -0.22 | 78.4 | 83.5 | 110.6 | Functions in nuclear protein import, either in association with an adapter protein. |
| *Tpr* (translocated promoter region) | -0.89 | -0.21 | -0.19 | 120.9 | 133.3 | 178.3 | Implicated in nuclear protein import. Plays a role in the mitotic spindle checkpoint. |
| *Snx14* (sorting nexin 14) | -0.85 | -0.20 | -0.55 | 58.6 | 72.3 | 37.3 | May be involved in several stages of intracellular trafficking (By similarity). |
| *Xpo1* (exportin 1, CRM1 homolog (yeast)) | -0.83 | -0.12 | -0.03 | 73.8 | 80.1 | 52.0 | Mediates the nuclear export of cellular proteins and RNAs. |
| **Genes involved in protein folding and degradation** | | | | | | | |
| *Pja2* (praja 2, RING-H2 motif containing) | -1.32 | -0.14 | -0.05 | 25.1 | 23.7 | - | Has E2-dependent E3 ubiquitin-protein ligase activity. Responsible for ubiquitination of cAMP-dependent protein kinase. |
| *Dnaja1* (DnaJ (Hsp40) homolog, subfamily A, member 1) | -1.09 | -0.07 | 1.17 | 34.4 | 69.9 | 40.5 | Cochaperone of HSP70s in protein folding and mitochondrial protein import. Loss of DNAJA1 in mice led to severe defects in spermatogenesis (Terada et al. 2005). |
| *Fbxo22* (F-box protein 22) | -1.04 | -0.12 | -0.05 | 18.3 | 35.0 | - | Substrate-recognition component of the SCF (SKP1-CUL1-F-box protein)-type E3 ubiquitin ligase complex |
| *Cct6a* (chaperonin containing Tcp1, subunit 6a (zeta)) | -0.86 | -0.23 | -0.04 | 18.4 | 37.1 | 27.4 | A molecular chaperone that is a member of the chaperonin containing TCP1 complex (CCT). The complex folds various proteins, including actin and tubulin. |
| *Trip12* (thhyroid hormone receptor interactor 12) | -0.85 | -0.07 | 0.10 | 70.4 | 74.9 | 78.6 | Probable E3 ubiquitin-protein ligase. |
| *Ppil4* (peptidylprolyl isomerase (cyclophilin)-like 4) | -0.84 | -0.19 | 0.23 | 59.8 | 66.8 | 83.2 | An important role in protein folding. |
| **Genes involved in mobile elements silencing** | | | | | | | |
| *Tdrd1* (tudor domain containing 1) | -1.57 | -0.40 | 0.42 | 23.8 | 48.1 | - | Plays a central role during spermatogenesis by participating to the repression transposable elements (Chuma et al. 2006). |
| *Piwil2* (piwi-like homolog 2 (Drosophila)) | -1.20 | -0.22 | -0.04 | 58.3 | 69.8 | - | Plays a central role during spermatogenesis by participating to the repression transposable elements, involved in translation regulation. Interacts with Tdrd1 (Kuramochi-Miyagawa et al. 2004; Wang et al. 2009). |
| *Mov10l1* (Moloney leukemia virus 10-like 1. homolog (mouse)) | -1.07 | -0.32 | 0.23 | 60.8 | 94.5 | - | Putative RNA helicase, essential for silencing retrotransposons in the mouse male germline (Frost et al. 2010; Zheng et al. 2010). |

*FDR > 0.125
