# Supplementary material for: Impact of heat shock transcription factor 1 on global gene expression profiles in cells which induce either cytoprotective or pro-apoptotic response following hyperthermia
Source: BMC Genomics. 2013 Jul 8;14:456. doi: 10.1186/1471-2164-14-456 (PMC3711851; doi:10.1186/1471-2164-14-456)
Supplement: Additional file 17: Table S8 — Characteristics of primers used in RT-PCR analyses. Available at: https://mynotebook.labarchives.com/share/HSF1%2520in%2520SC%2520and%2520HEP/NDQuMnwxMjY2MS8zNC00Mi9UcmVlTm9kZS8yMDM3OTUxNzR8MTEyLjI. [file 1471-2164-14-456-S17.docx]

**Table S8**. Characteristics of primers used in RT-PCR analyses

| **Gene symbol** | **NCBI reference sequence** | **Primers sequences** | **Product lenght [bp]** | **Position in the reference sequence** |
| --- | --- | --- | --- | --- |
| *Gapdh* | NM_008084 | F: tgtcttcaccaccatggaga  R: cggccatcacgccacagctt | 300 | 344 - 643 |
| *Actb* | NM_007393 | F: ggacttcgagcaagagatgg  R: agcactgtgttggcgtacag | 234 | 742 - 975 |
| *18s rRNA* | NR_003278 | F: gttggtggagcgatttgtctgg  R: gcagccccggatctaagg | 178 | 1345 - 1522 |
| *BC048609* | NM_001111317 | F: gctgtgttatgcttccctcc  R: tcctctcggtaattgatggc | 320 | 127 - 446 |
| *1700007E05Rik* | XR_002317 | F: ccctccagtccttgtttgaa  R: aagtgaccatggtgctttcc | 330 | 1906 - 2235 |
| *Atf3* | NM_007498 | F: aaagaaggaacattgcagagc  R: gataaggcctaaatcctgga | 260 | 769 - 1028 |
| *Bag3* | NM_013863 | F: attgatgtcccaggtcaagt  R: ccccaagttactgcataccaa | 333 | 1786 - 2118 |
| *Celf1*  (2 transcript variants) | NM_017368  NM_198683 | F: ggcttaaagtgcagctcaaacg  R: cagcatcaaaggtcaacacaagg | 221 | 1758 - 1978  1606 - 1826 |
| *Dapl1* | NM_029723 | F: aacaagagatgggcgttttg  R: tggctgtgttttctgtcctg | 258 | 145 - 402 |
| *Egr1* | NM_007913 | F: tgatcctctattttgtgatg  R: aagctcagctcagccctctt | 375 | 2413 - 2787 |
| *Fos* | NM_010234 | F: ggaattaacctggtgctgga  R: ccacatgtcgaaagacctca | 356 | 1658 - 2013 |
| *Hsp90aa1* | NM_010480 | F: ggcatgaaaactaaggggaag  R: agggttgttctcgggacttt | 315 | 2443 - 2757 |
| *Hsp90ab1* | NM_008302 | F: ctgctctgctctcctctggt  R: attctgtgggaggggatctt | 339 | 2077 - 2415 |
| *Hspa1a*  *Hspa1b* | NM_010479  NM_010478 | F: ccatccagagacaagcgaag  R: cgtttagaccggcgatcac | 699 | 38 - 736 |
| *Hspa8* | NM_031165 | F: aggcaagatcaatgatgagga  R: acttgttttgggtccctgtg | 313 | 1758 - 2070 |
| *Hsph1* | NM_013559 | F: agttgcagccaaaaaccagca  R: ccagcacagaccttcgctc | 326 | 386 - 711 |
| *Jun* | NM_010591 | F: gaacttgactggttgcgaca  R: aggtccatgcagttcttggt | 251 | 2016 - 2266 |
| *Mov10l1* | NM_031260 | F: gggtgtgatctgcctcctgaa  R: gaggctcaatggctccctgtt | 190 | 3650 - 3839 |
| *Phlda1* | NM_009344 | F: caacagctccactcctaccc  R: gcttcctgcaactgtgatga | 394 | 1149 - 1542 |
| *Pyhin1* | NM_175026 | F: ggaggtctacggacgactga  R: tgccatttcctaaaccaaaga | 306 | 1433 - 1738 |
| *Rsad2* | NM_021384 | F: gtcctgtttggtgcctgaat  R: actggaccttgctcctctga | 449 | 894 - 1342 |
| *Socs3* | NM_007707 | F: ggcacatggcacaagcacaa  R: aatcaaagcgcaaacaagttc | 364 | 1311 - 1674 |
| *Spo11*  (3 transcript variants) | NM_012046  NM_001083960  NM_001083959 | F: cctaccagcccctctggaaaa  R: tcaagcaggccaacagaaagc | 195 | 1098 – 1292  984 – 1178  1023 - 1217 |
| *Tdrd1*  (4 transcript variants) | NM_001002238  NM_001002240  NM_001002241  NM_031387 | F: tcctcttaaacaatccaaccaacca  R: gcacagcggagaggctaaaa | 206 | 3540 – 3745  3513 - 3718  3813 - 4018  3673 - 3878 |
| *Zfp36l1* | NM_007564 | F: cccctaccttggacaactca  R: gcgtggttaagtgggctatg | 301 | 1091 - 1391 |
