# Supplementary material for: Impact of heat shock transcription factor 1 on global gene expression profiles in cells which induce either cytoprotective or pro-apoptotic response following hyperthermia
Source: BMC Genomics. 2013 Jul 8;14:456. doi: 10.1186/1471-2164-14-456 (PMC3711851; doi:10.1186/1471-2164-14-456)
Supplement: Additional file 18: Table S9 — Characteristics of primers used in ChIP-PCR analyses. Available at: https://mynotebook.labarchives.com/share/HSF1%2520in%2520SC%2520and%2520HEP/NDIuOXwxMjY2MS8zMy0zOC9UcmVlTm9kZS80NTMxNzgxMTR8MTA4Ljk. [file 1471-2164-14-456-S18.docx]

**Table S9**. Characteristics of primers used in ChIP-PCR analyses

| **Gene symbol (Entrez Gene ID)** | **HSEs position*** | **Primers sequences** | **Product lenght [bp]** | **Position of the amplified sequence*** |
| --- | --- | --- | --- | --- |
| *Dnaja1* (15502) | (-195) – (-181)  (+1) – (+15) | F cactctggttcctcgcaata  R accgagcgttctggaaagt | 247 | (-225) – (+22) |
| *Hsph1* (15505) | (-151) – (-134) (-87) – (-70) | F ctgtcaccatggcaactcag  R ccaatcgctcagccttatgt | 175 | (-211) – (-37) |
| *Hspa1a* (193740)  *Hspa1b* (15511) | (-114) – (-97) | F: aactccgattactcaagggaggc  R: gattctgagtagctgtcagcg | 184 | (-157) – (+27) |
| *Phlda1* (21664) | (-22) – (-5) | F taatgcccgctatggaactt  R ctggaagcaggttctggaaa | 261 | (-260) – (+1) |

*against transcription start site (+1) according to DBTSS
